# Supplementary figures and images for: Caspase 3 and caspase 7 promote cytoprotective autophagy and the DNA damage response during non-lethal stress conditions in human breast cancer cells
Source: PLoS Biol. 2025 Feb 21;23(2):e3003034. doi: 10.1371/journal.pbio.3003034 (PMC11882052; doi:10.1371/journal.pbio.3003034)

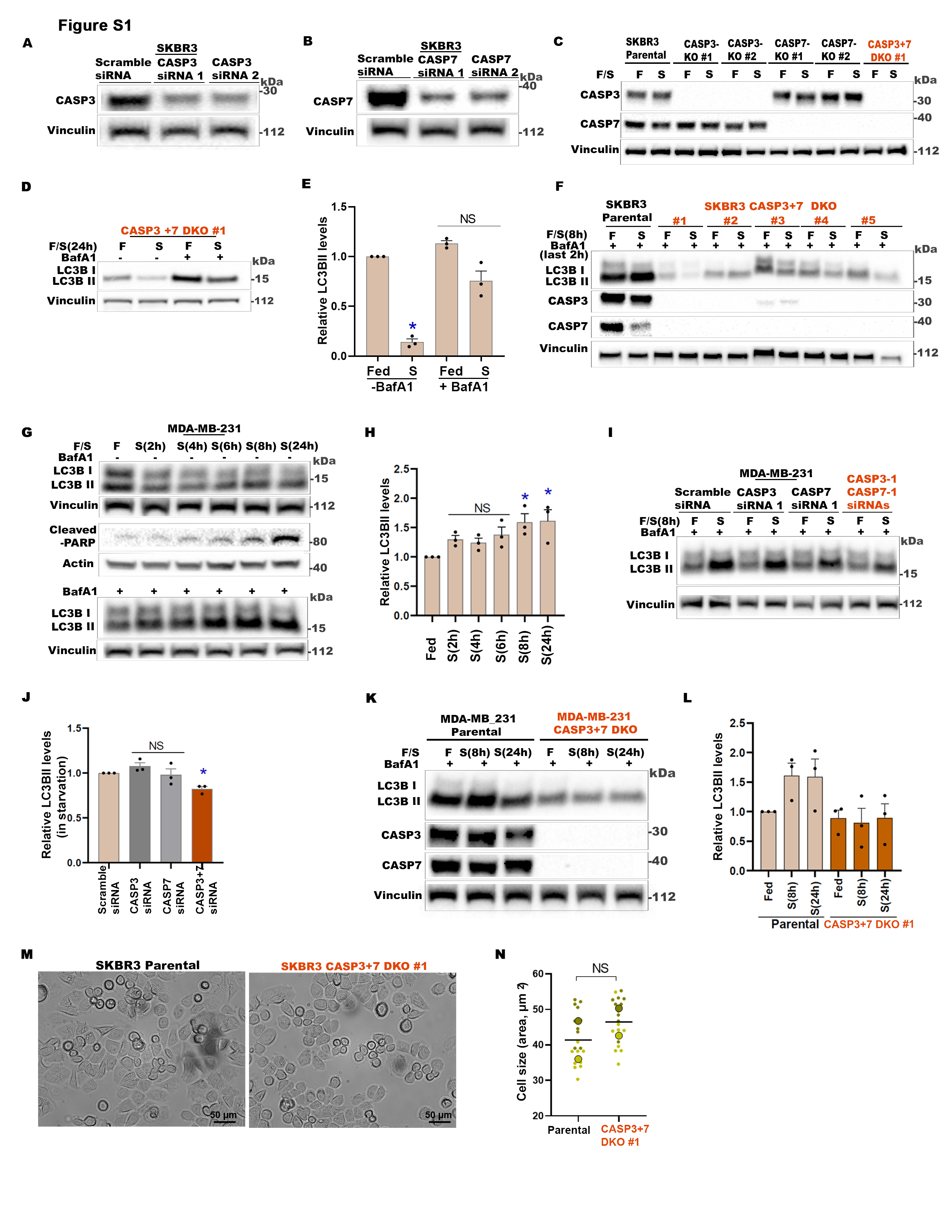

Supplement: S1 Fig — (A, B) Representative western blots showing levels of CASP3 or CASP7 following treatment with scramble-siRNA, CASP3 siRNA or CASP7 siRNAs used in experiments. (C) Representative western blots showing levels of CASP3 and CASP7 in knockout lines of CASP3 and/or CASP7 in SKBR3 cells, cultured in fed conditions (F; fresh DMEM) or subjected to amino acid starvation (S) in EBSS for 8 h. (D) Representative western blots of indicated proteins from CASP3 + 7 DKO SKBR3 cells cultured in fed conditions (fresh DMEM) or subjected to amino acid starvation in EBSS for 24 h, in the absence or presence of BafA1(50 nM) for the final 2 h. (E) Quantification of LC3B-based autophagy flux shown in (D). The levels of LC3BII were normalized to loading control and shown relative to the fed (fresh DMEM) in the absence of BafA1. (F) Representative western blots of indicated proteins from SKBR3 parental and multiple CASP3 + 7 DKO cell lines (CRISPR-Cas9 mediated), cultured in fed conditions (fresh DMEM) or subjected to amino acid starvation in EBSS for 8 h. BafA1 (50 nM) was added for the final 2 h of culture. (G) Representative western blots of indicated proteins from MDA-MB-231 cells cultured in fed conditions (fresh DMEM) or subjected to amino acid starvation in EBSS for various time periods, in the absence (top) or presence (bottom) of BafA1(50 nM) for the final 2 h. (H) Quantification of LC3B-based autophagy flux shown in (G). The levels of LC3BII (in the presence of BafA1) were normalized to loading control and shown relative to the fed (fresh DMEM) control. (I) Representative western blots of indicated proteins from MDA-MB-231 cells transfected with scramble, CASP3 and/or CASP7 siRNAs (48 h) and then continued to be cultured in fed conditions (fresh DMEM) or starved in EBSS for 8 h. BafA1 (50 nM) was added for the final 2 h. (J) Quantification of LC3BII-based autophagy flux shown in (I). The levels of LC3BII in starved cells were normalized to loading control and shown relative to the s [file pbio.3003034.s001.tif]

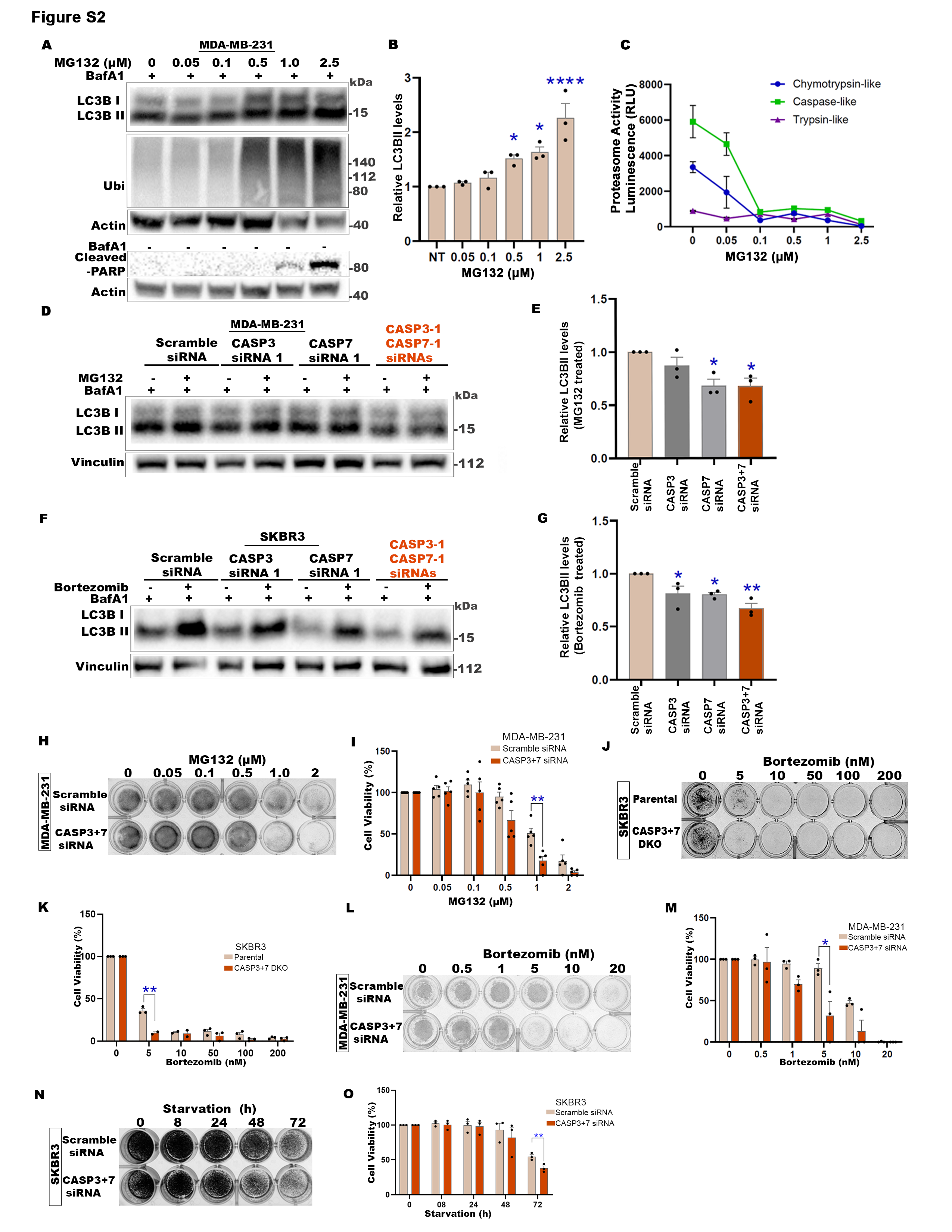

Supplement: S2 Fig — (A) Representative western blots of indicated proteins from MDA-MB-231 cells treated with proteasome inhibitor (MG132) at increasing dosage for 24 h in the presence (top) or absence (bottom) of BafA1(50 nM) for the final 2 h. (B) Quantification of LC3B-based autophagy flux in MDA-MB-231 shown in (A). The levels of LC3BII were normalized to loading control and shown relative to the untreated control (NT). (C) Graph showing proteasome activity in MDA-MB-231 cells in response to increasing concentrations of MG132 as depicted by chymotrypsin-like, caspase-like, and trypsin-like activity measured by the Proteasome glow assay kit. (D) Representative western blots of indicated proteins from MDA-MB-231 cells transfected with scramble, CASP3 and/or CASP7 siRNAs (48 h) and continued to be cultured in vehicle DMSO or MG132 (0.5 μM) treated fresh DMEM for 24 h. BafA1(50 nM) was added for the final 2 h. (E) Quantification of LC3B-based autophagy flux in proteasome inhibitor (MG132) treated MDA-MB-231 cells shown in (D). The levels of LC3BII in MG132-treated cells were normalized to loading control and shown relative to the MG132-treated scramble-siRNA control. (F) Representative western blots of indicated proteins from SKBR3 cells transfected with scramble, CASP3 and/or CASP7 siRNAs (48 h) and continued to be cultured in vehicle DMSO or Bortezomib- (1 nM) treated fresh DMEM for 24 h. BafA1 (50 nM) was added for the final 2 h. (G) Quantification of LC3B-based autophagy flux in proteasome inhibitor- (Bortezomib) treated SKBR3 cells shown in (F). The levels of LC3BII in Bortezomib-treated cells were normalized to loading control and shown relative to the Bortezomib-treated scramble-siRNA control. (H–M) Representative images of crystal violet assay plates and quantification of percentage of cell viability. The indicated siRNA transfected cells were grown for 2 days or the Parental and DKO cells were grown in normal media for 2 days and treated with indicated concentrations of protea [file pbio.3003034.s002.tif]

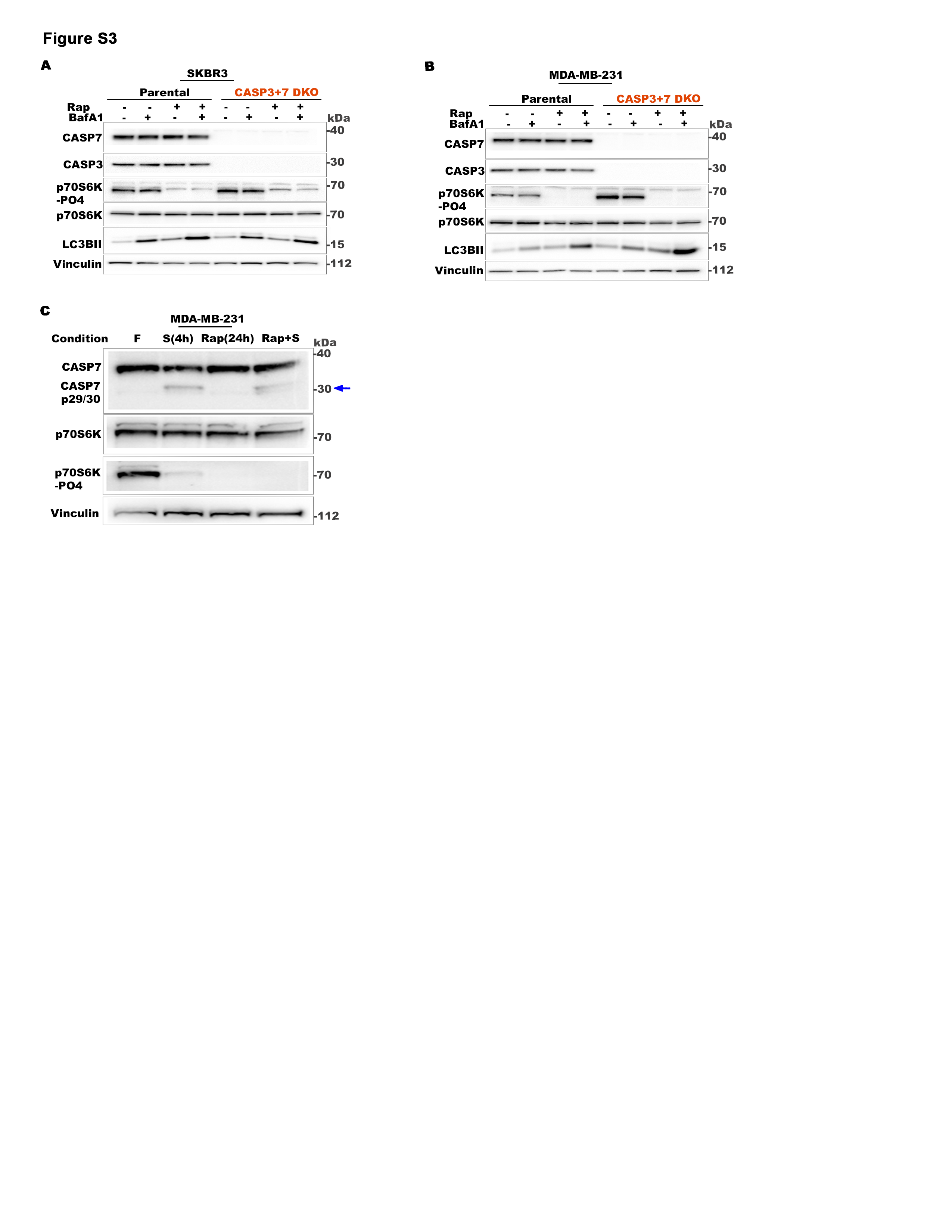

Supplement: S3 Fig — (A, B) Representative western blots of indicated proteins from SKBR3 (A) or MDA-MB-231 (B) parental and CASP3 + 7 DKO cells untreated or treated with mTOR inhibitor rapamycin (Rap) 10 nM for 24 h in the absence or presence of BafA1 (50 nM) for the final 2 h. The levels of phosphorylated and unphosphorylated p70S6K were used as markers for mTOR activity. (C) Representative western blots showing the effects of rapamycin on the formation of CASP7-p29/p30 bands in MDA-MB-231 cells. Cells grown in normal culture media for 4 days were continued to be cultured in fed (fresh DMEM) conditions, starved in EBSS for 4 h, treated with 10 nM rapamycin (Rap) for 24 h or subjected to both starvation and Rap treatment for 24 h. The p70S6K and p70S6K-PO4 immunolabeling was used as an mTOR activity reporter. In each n = 2 independent experiments. (TIF) [file pbio.3003034.s003.tif]

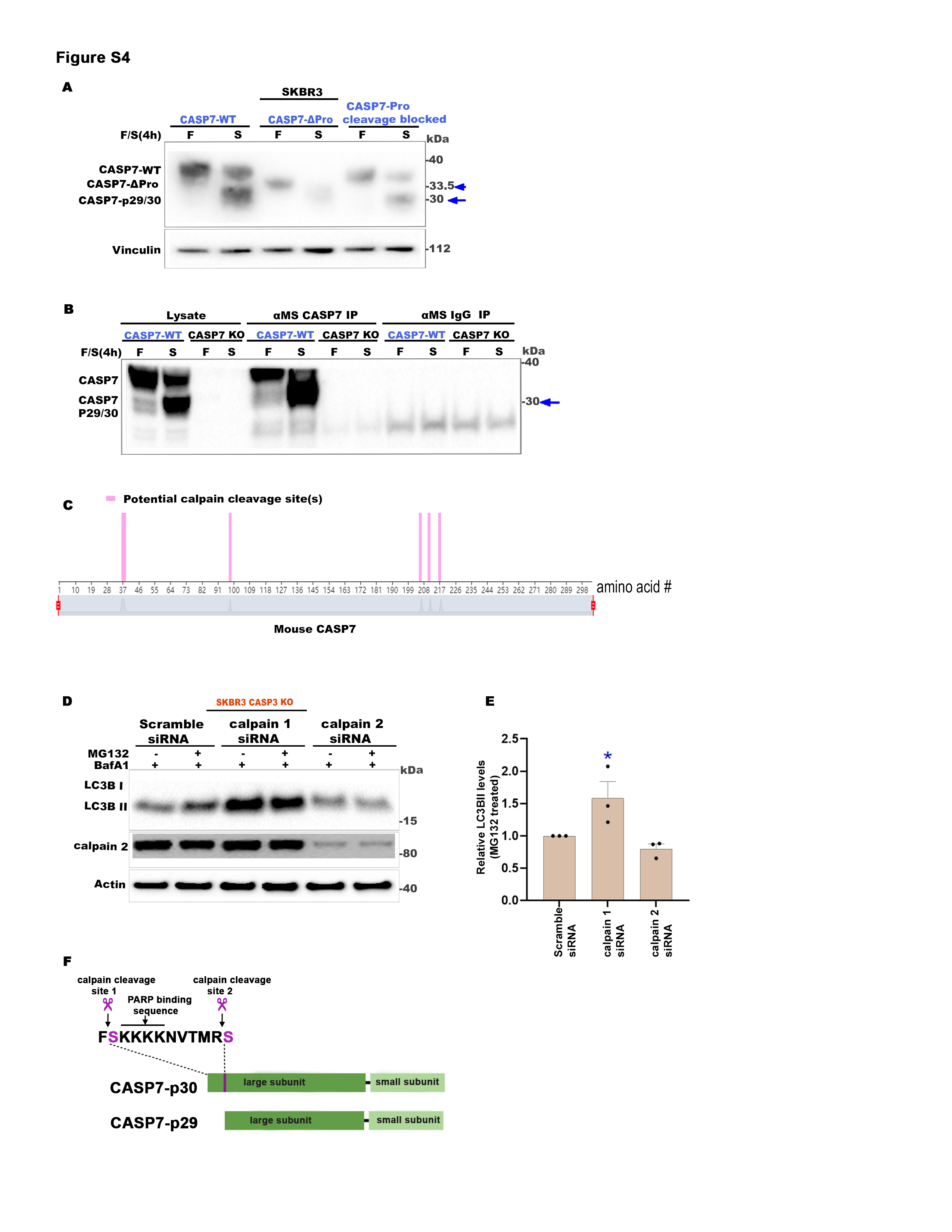

Supplement: S4 Fig — (A) Representative western blot showing CASP7 immunolabeling in CASP3 + 7 DKO SKBR3 cells stably expressing CASP7-WT, CASP7 ΔPro, and CASP7 prodomain cleavage blocked constructs cultured in fed conditions (fresh DMEM) or starved in EBSS for 4 h. Arrowhead indicates CASP7-ΔPro fragment (33.5 kDa) and arrow indicates CASP7-p29/30 fragments. n = 1. (B) Representative western blot showing immunoprecipitation of CASP7-p29/30 fragment(s) for Edman sequencing. CASP7 KO SKBR3 cells stably expressing CASP7-WT construct were cultured in fed conditions (fresh DMEM) or starved in EBSS for 2 h prior to immunoprecipitation with CASP7 antibody. Lysate are from CASP7-WT expressing and CASP7-KO cells. IP with anti-mouse CASP7 and anti-mouse IgG antibodies are shown. n = 2 independent experiments. (C) A schematic showing mouse CASP7 with predicted calpain cleavage sites identified by the Deepcalpain algorithm. (D) Representative western blots of indicated proteins from CASP3 KO SKBR3 cells transfected with scramble, calpain 1, or calpain 2 siRNAs (48 h) and then continued to be cultured in vehicle DMSO or MG132 (0.5 μM) in fresh DMEM for 24 h and with BafA1(50 nM) in the final 2 h. (E) Quantification of LC3B-based autophagy flux in MG132-treated cells shown in (D) relative to MG132-treated scramble-siRNA control. (F) Schematic showing the amino acid sequence between two non-canonical cleavage sites in CASP7. PARP binding site is shown. In graph (E), data are shown as mean ± SEM. n = 3 independent experiments. *P < 0.05, **P < 0.01, ***P < 0.001, ****P < 0.0001, NS, not significant, with one-way ANOVA with Dunnett’s post-test. The numerical data presented in this figure can be found in S2 Data. (TIF) [file pbio.3003034.s004.tif]

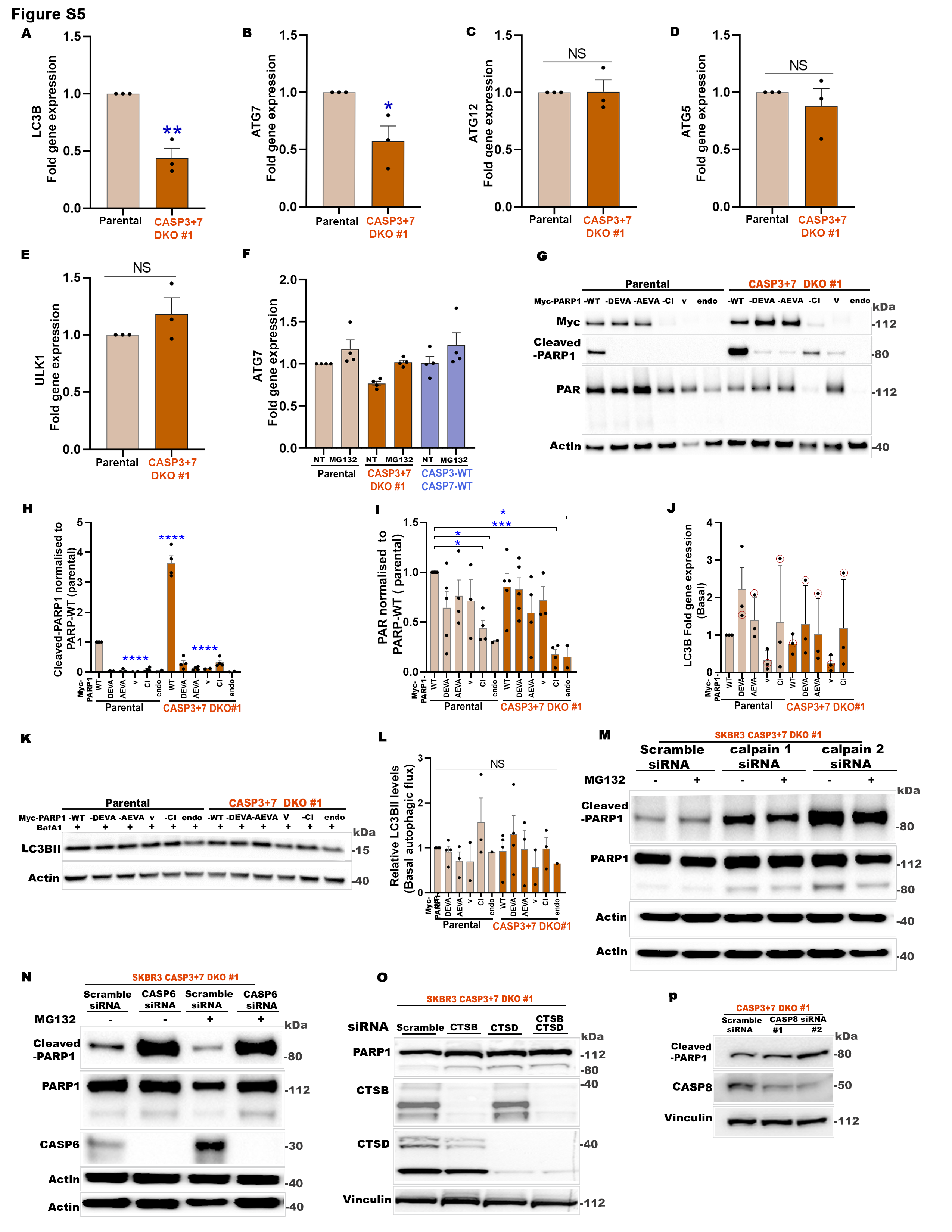

Supplement: S5 Fig — (A–E) Reverse transcription polymerase chain reaction (RT-qPCR) analyses of key ATG gene expression in SKBR3 parental or CASP3 + 7 DKO cells under well-fed conditions. (F) RT-qPCR analyses of ATG7 in SKBR3 parental, CASP3 + 7 DKO, or CASP3 + 7 DKO cells re-expressing CASP3 + 7-WT constructs, treated with vehicle DMSO (NT) or 0.5 μM of MG132 for 24 h. (G) Representative western blots of indicated proteins from SKBR3 parental or CASP3 + 7 DKO cells, transiently transfected with indicated Myc-tagged PARP constructs (PARP1-WT, PARP1-DEVA, PARP1-AEVA, or catalytically inactive PARP1-CI) or vector control (v) for 48 h. endo = endogenous PARP1. (H-I) Quantification of cleaved-PARP1 (H) and PAR levels (I) shown in (G). (J) RT-qPCR analyses of LC3B transcripts in basal conditions in SKBR3 parental or CASP3 + 7 DKO cells transiently transfected with indicated Myc-tagged PARP constructs (PARP1-WT, PARP1-DEVA, PARP1-AEVA, or PARP1-CI) or vector control (v). Data of one replicate with relatively high LC3B levels is circled in red. (K) Representative western blots of indicated proteins from SKBR3 parental or CASP3 + 7 DKO cells, transiently transfected with indicated Myc-tagged PARP constructs (PARP1-WT, PARP1-DEVA, PARP1-AEVA, or PARP1-CI) or vector control (v) for 48 h and with BafA1 (50 nM) in the final 2 h. endo = endogenous PARP1 (L) Quantification of LC3B-based autophagic flux in basal conditions, shown in (K). (M-P) Representative western blots of indicated proteins from SKBR3 cells transfected with scramble, calpain 1, calpain 2, CASP6, cathepsin B (cathB), cathepsin D (cathD) or CASP8 siRNAs (48 h) and treated with vehicle DMSO or MG132 (0.5 μM) for 24 h (M and N) or grown in untreated media (in O and P). Each with at least n = 2 independent experiments. In graphs, unless otherwise noted, all data are shown as mean ± SEM. n = 3 or more independent experiments. *P < 0.05, **P < 0.01, ***P < 0.001, ****P < 0.0001, NS, not significant. A-E with Student T test (unpaired). In [file pbio.3003034.s005.tif]

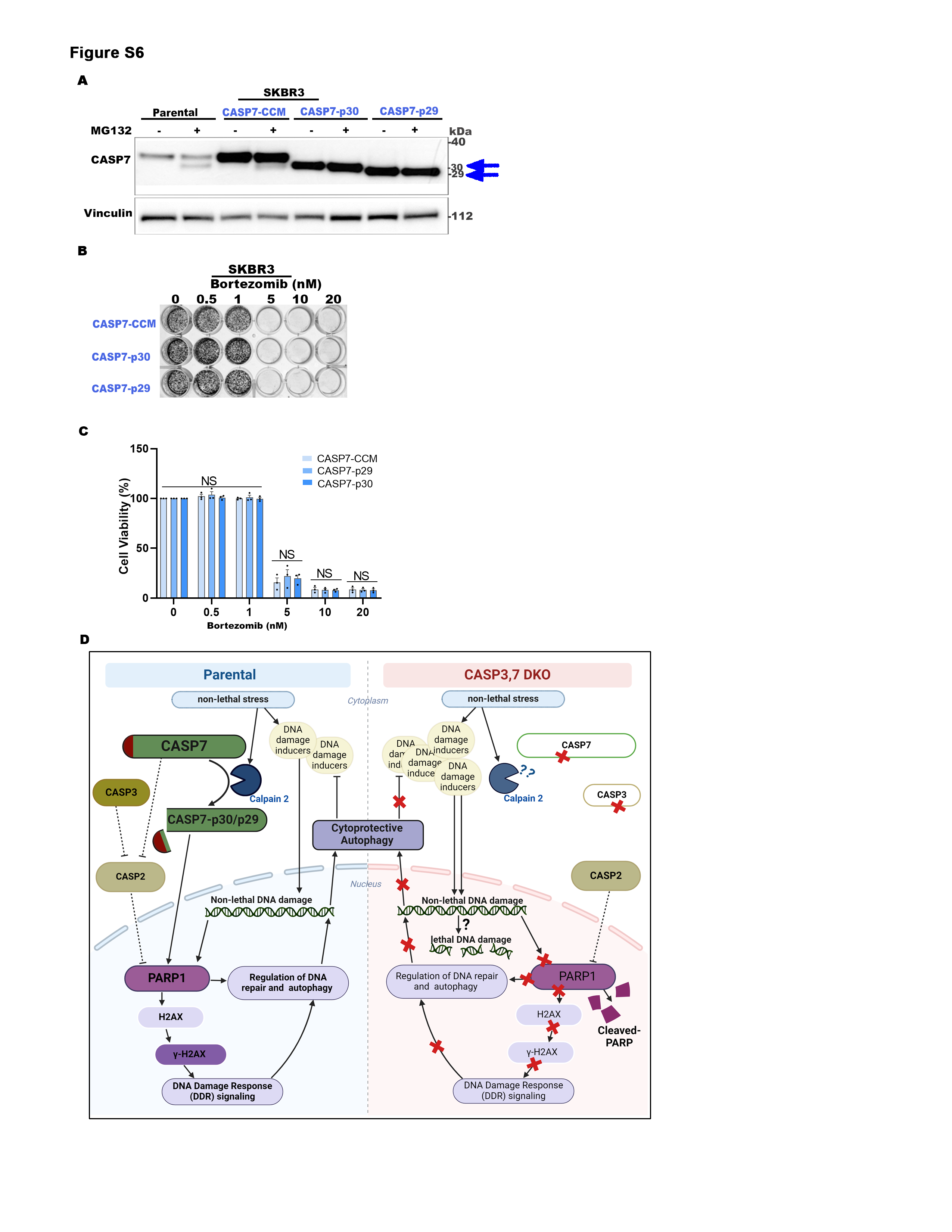

Supplement: S6 Fig — (A) Representative western blot of CASP7 immunolabeling from SKBR3 parental or CASP3 + 7 DKO cells stably expressing CASP7-CCM, CASP7-p30 or CASP7-p29 constructs, treated with vehicle DMSO or proteasome inhibitor MG132 (0.5 μM) for 24 h. n = 2 independent experiments. (B) Representative images of crystal violet assay plates and quantification in cells expressing CASP7-CCM, CASP7-p30 or CASP7-p29 constructs. Cells were grown in standard media for 2 days and treated with indicated concentrations of proteasome inhibitor Bortezomib for 24 h and continued to grow in drug free media for another 3 days before subjected to crystal violet assay. (C) Quantification of cell viability data presented in (B). The percentage of stained (viable) cells at each concentration was normalized to respective untreated cells. Data are shown as mean ± SEM. n = 3 independent experiments. NS, not significant. Two-way ANOVA with Tukey’s post-test. (D) Graphical representation of current working model. The numerical data presented in this figure can be found in S2 Data. (TIF) [file pbio.3003034.s006.tif]

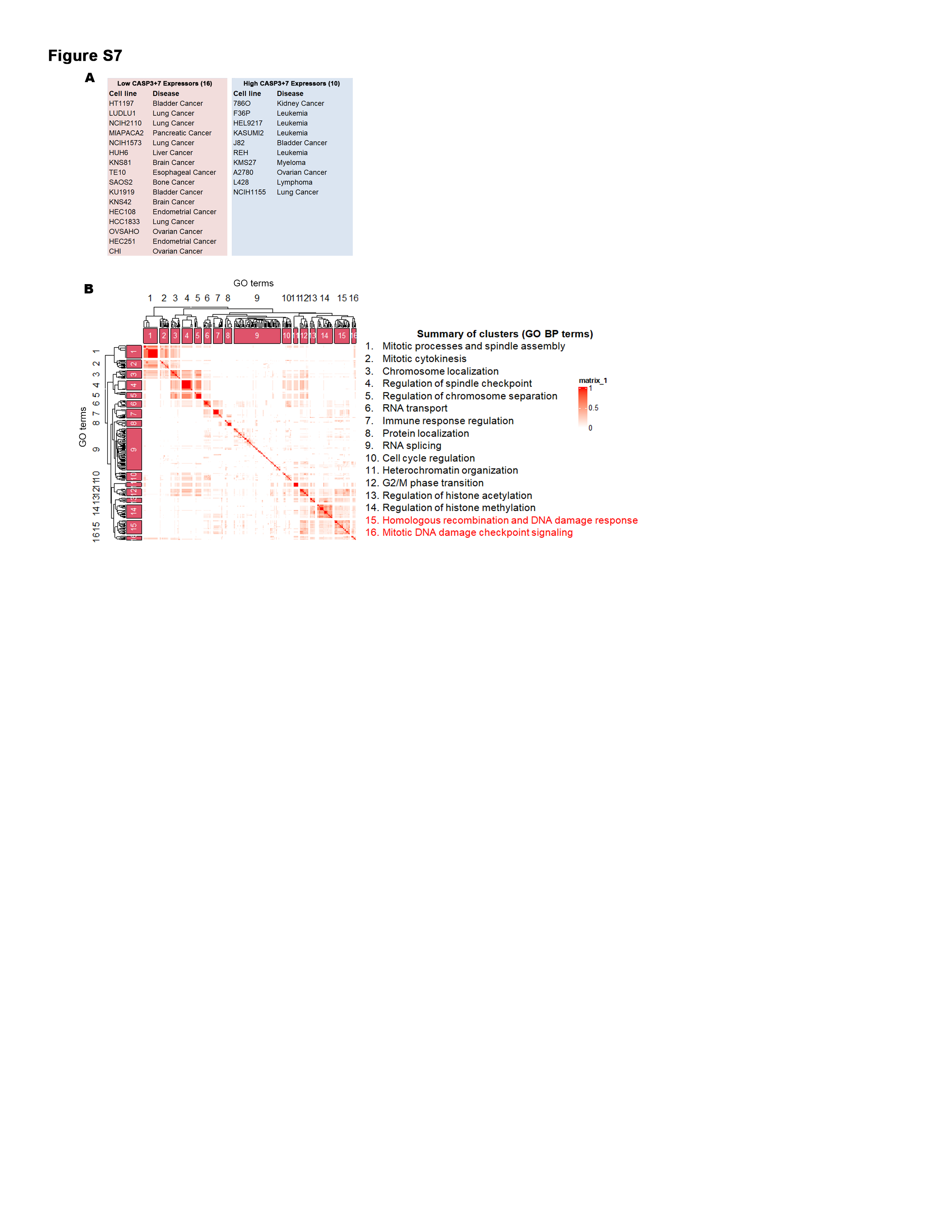

Supplement: S7 Fig — (A) Low and high levels of CASP3 and CASP7 expressing cancer cell lines identified and analyzed in Fig 7H and I. (B) Heatmap showing Jaccard index similarities between GO terms. Based on annotated GO biological processes of genes that were candidate co-essential or genetic interactors (unadjusted p-value < 0.05). GO terms were summarized into 16 distinct clusters using Jaccard index-based hierarchical clustering. The numerical data presented in this figure can be found in S2 Data. The code related to S7A and S7B Fig is publicly available in a GitHub repository (https://github.com/MarraLab/Caspase_GRETTA_analysis) and archived on Zenodo (https://doi.org/10.5281/zenodo.14722298) (TIF) [file pbio.3003034.s007.tif]
