# Supplementary material for: Caspase 3 and caspase 7 promote cytoprotective autophagy and the DNA damage response during non-lethal stress conditions in human breast cancer cells
Source: PLoS Biol. 2025 Feb 21;23(2):e3003034. doi: 10.1371/journal.pbio.3003034 (PMC11882052; doi:10.1371/journal.pbio.3003034)

Figure 1

A

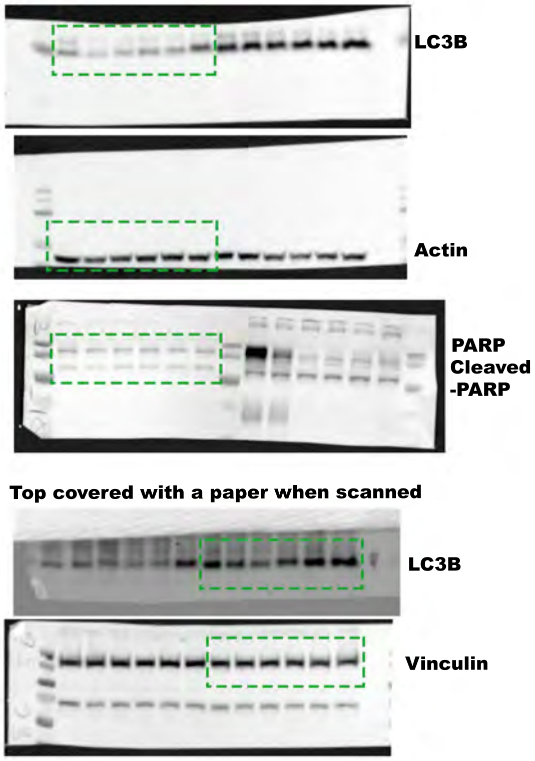

C

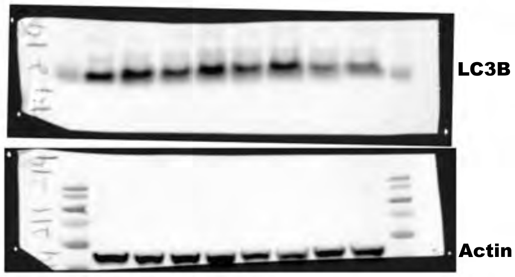

E

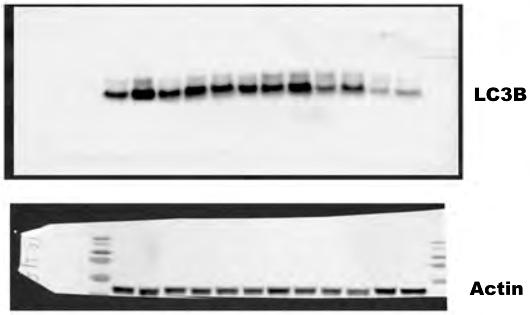

G

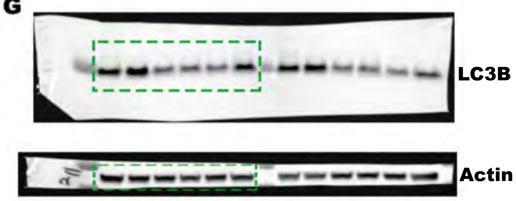

Figure 2

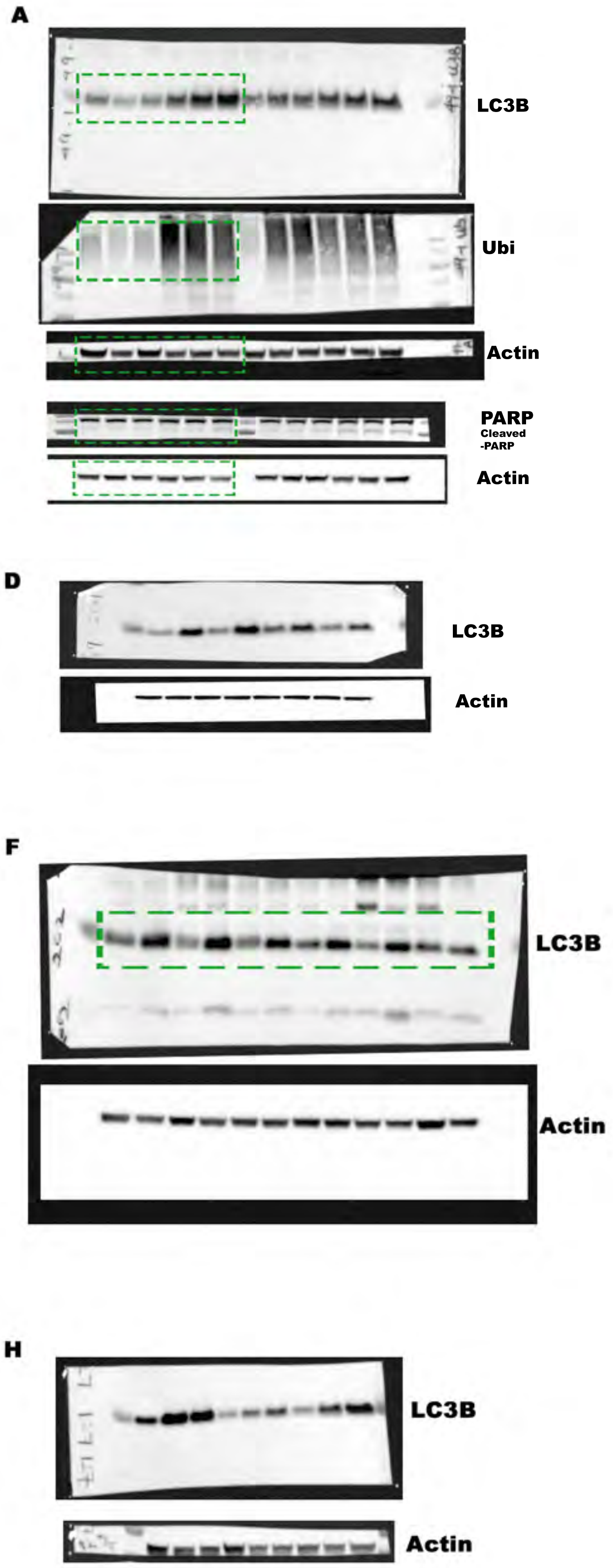
A
LC3B
Ubi
Actin
PARP
Cleaved
-PARP
Actin
D
LC3B
Actin
F
LC3B
Actin
H
LC3B
Actin

Figure 3

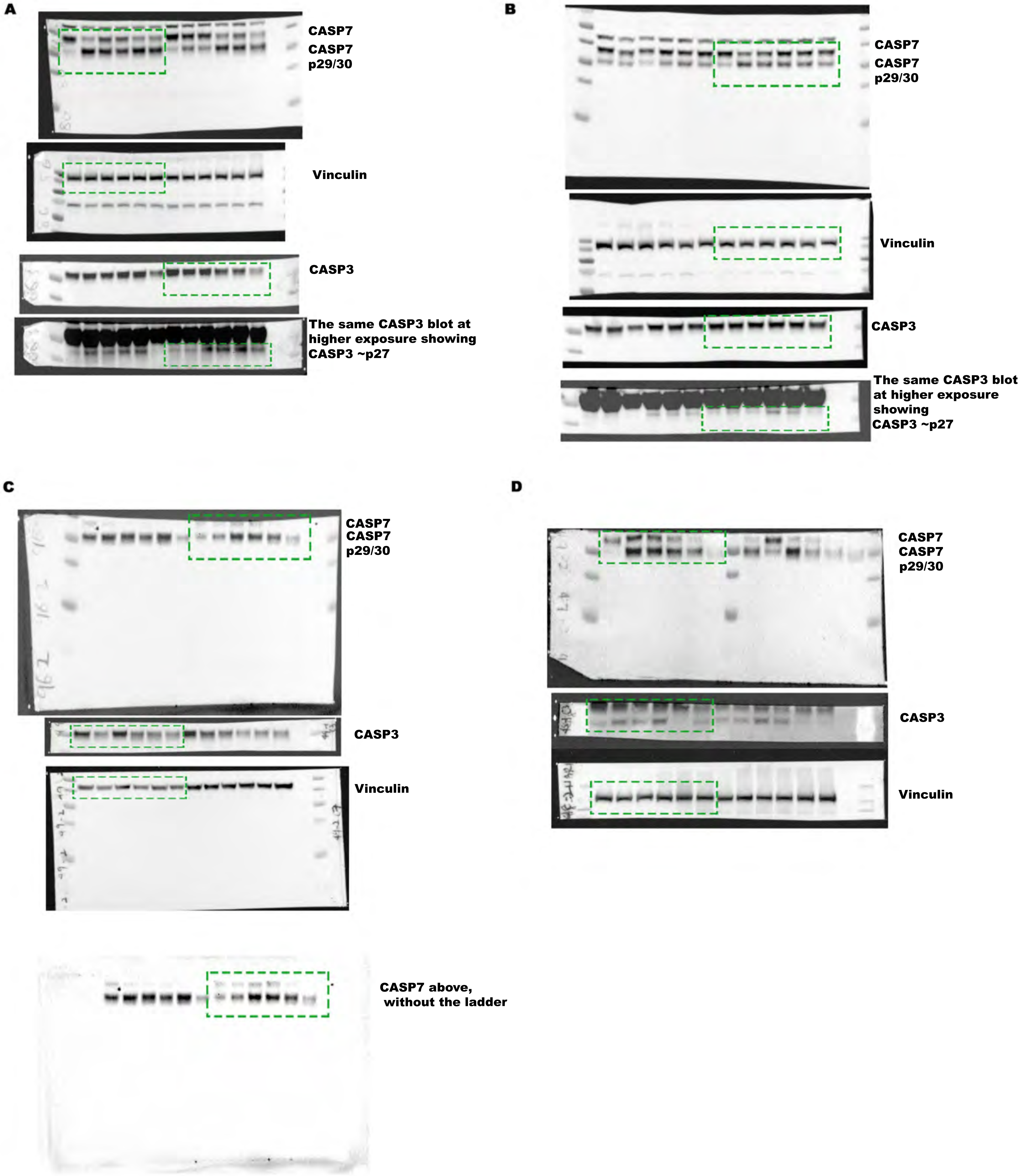
A
CASP7
CASP7 p29/30
Vinculin
CASP3
The same CASP3 blot at higher exposure showing CASP3 ~p27
B
CASP7
CASP7 p29/30
Vinculin
CASP3
The same CASP3 blot at higher exposure showing CASP3 ~p27
C
CASP7
CASP7 p29/30
CASP3
Vinculin
CASP7 above, without the ladder
D
CASP7
CASP7 p29/30
CASP3
Vinculin

Figure 3

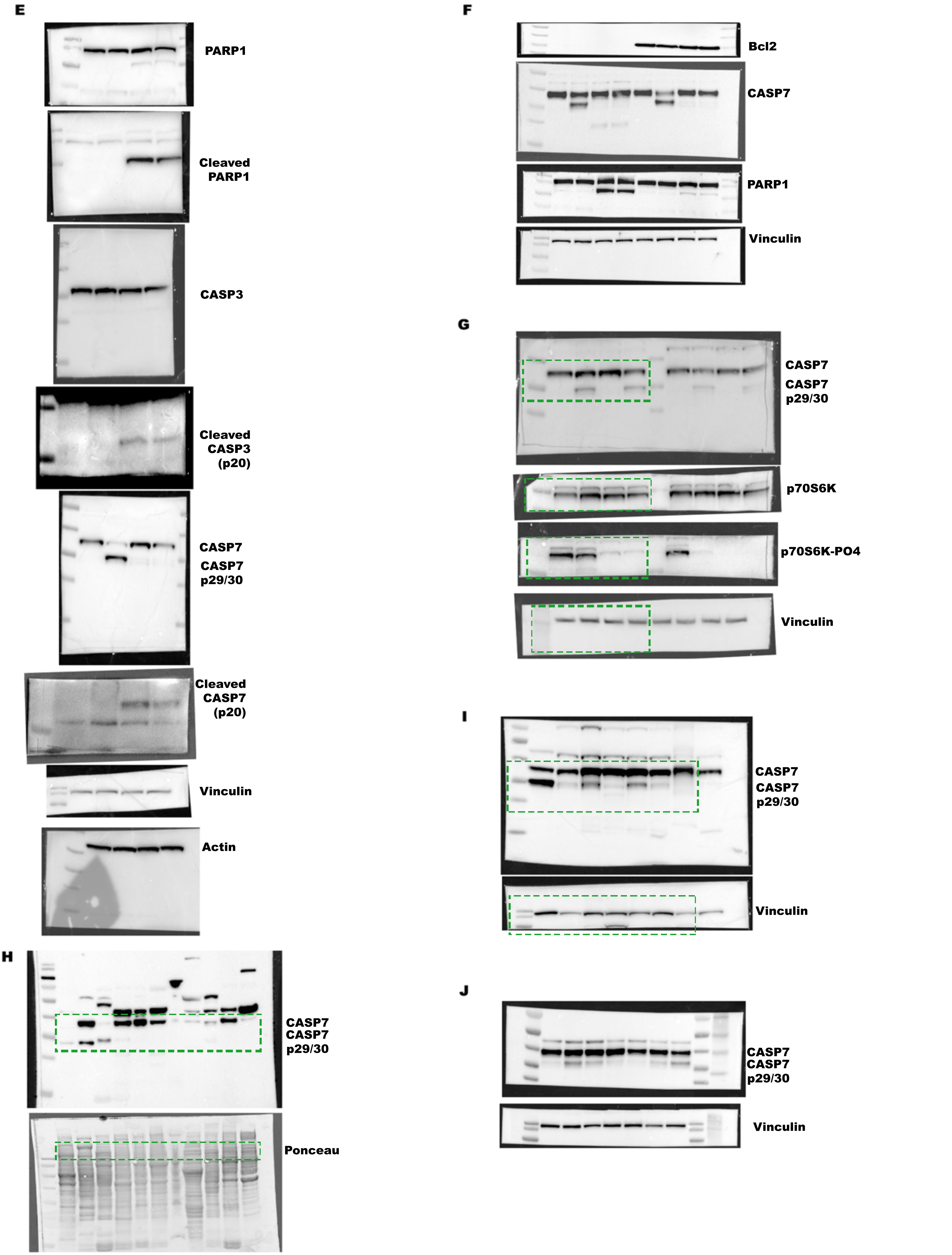
E
PARP1
Cleaved PARP1
CASP3
Cleaved CASP3 (p20)
CASP7
CASP7 p29/30
Cleaved CASP7 (p20)
Vinculin
Actin
F
Bcl2
CASP7
PARP1
Vinculin
G
CASP7
CASP7 p29/30
p70S6K
p70S6K-PO4
Vinculin
H
CASP7
CASP7 p29/30
Ponceau
I
CASP7
CASP7 p29/30
Vinculin
J
CASP7
CASP7 p29/30
Vinculin

Figure 4

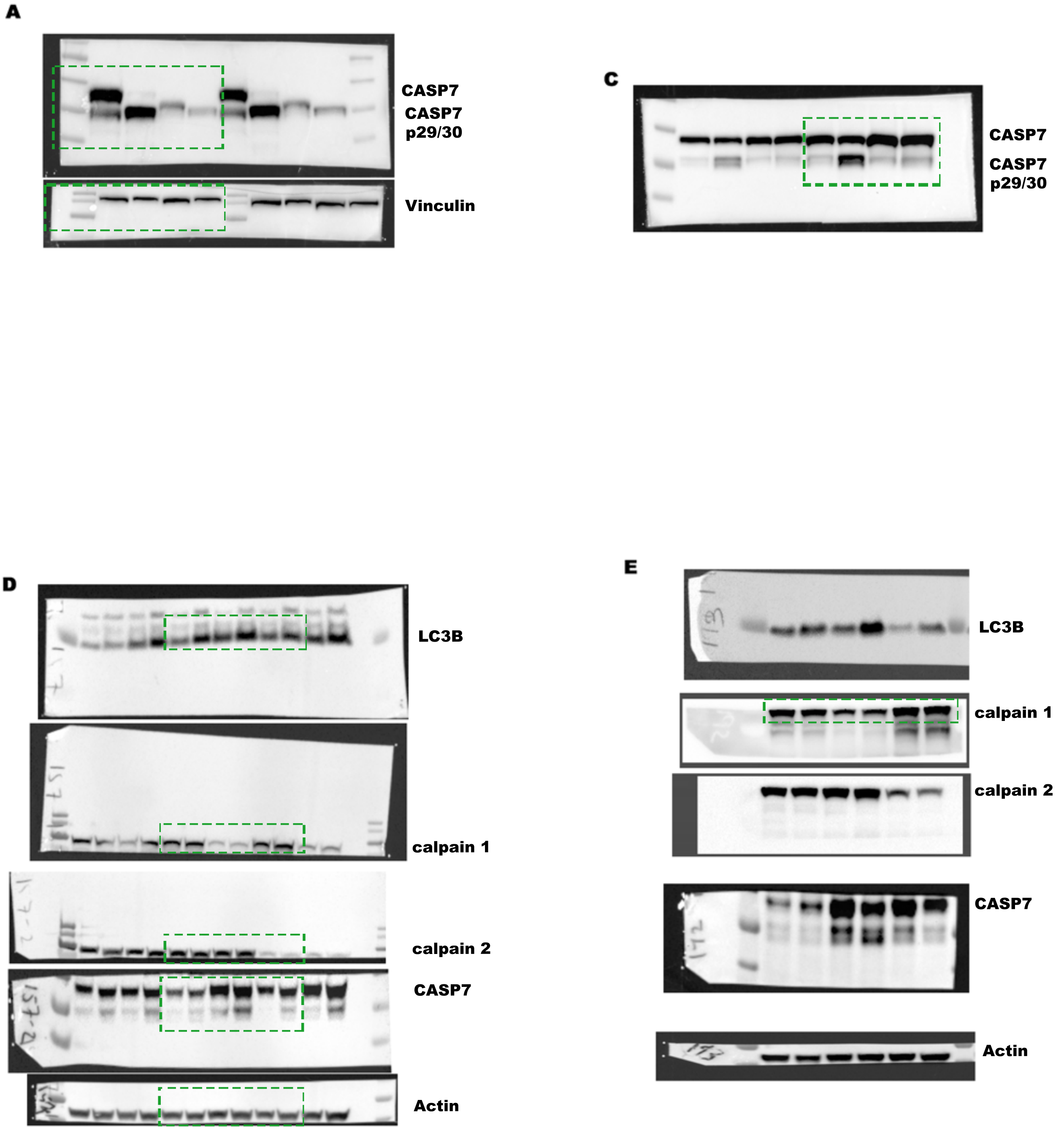
A
CASP7
CASP7
p29/30
Vinculin
C
CASP7
CASP7
p29/30
D
LC3B
calpain 1
calpain 2
CASP7
Actin
E
LC3B
calpain 1
calpain 2
CASP7
Actin

Figure 5

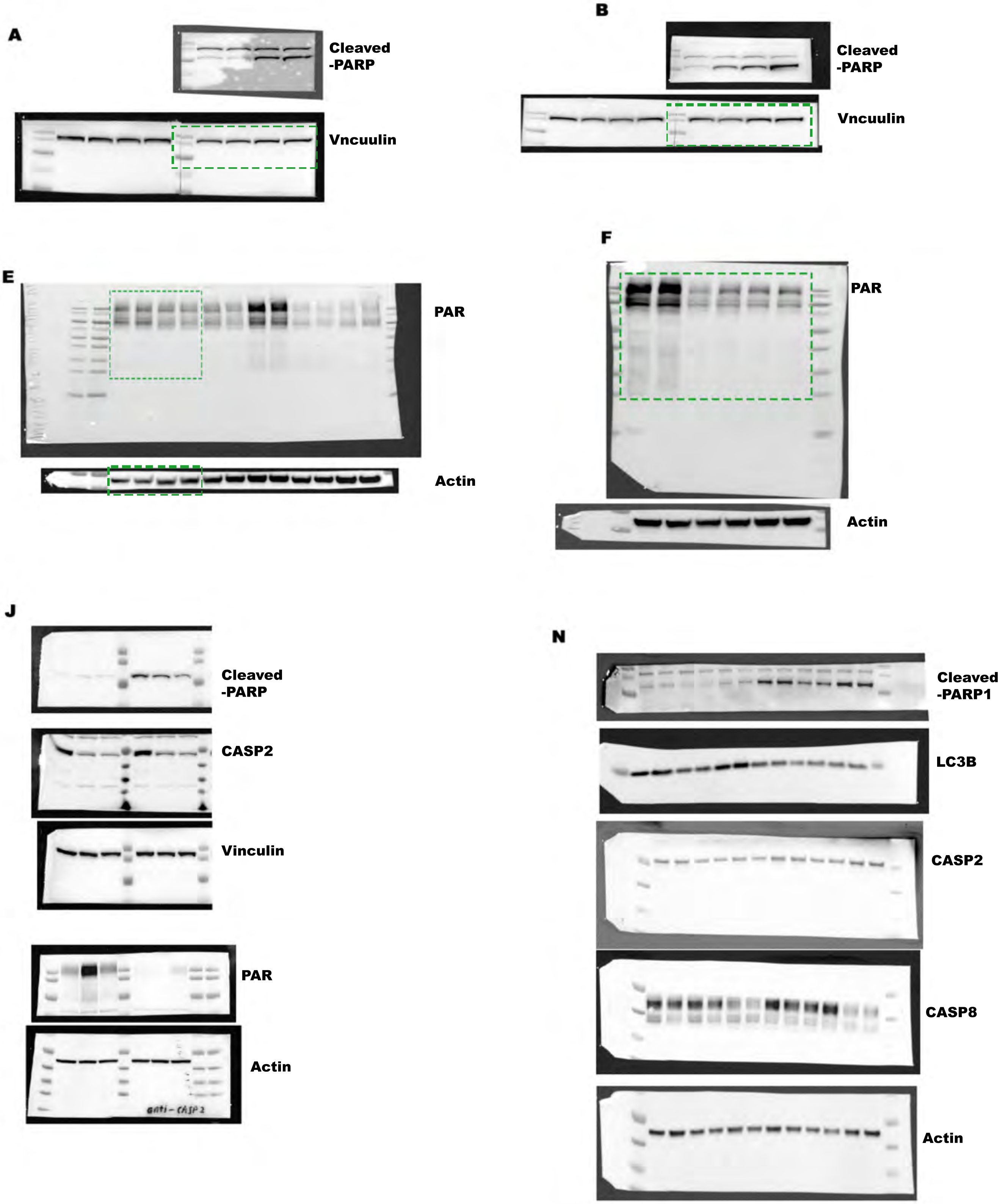
A
Cleaved
-PARP
Vncuulin
B
Cleaved
-PARP
Vncuulin
E
PAR
Actin
F
PAR
Actin
J
Cleaved
-PARP
CASP2
Vinculin
PAR
Actin
anti-CASP2
N
Cleaved
-PARP1
LC3B
CASP2
CASP8
Actin

Figure 6

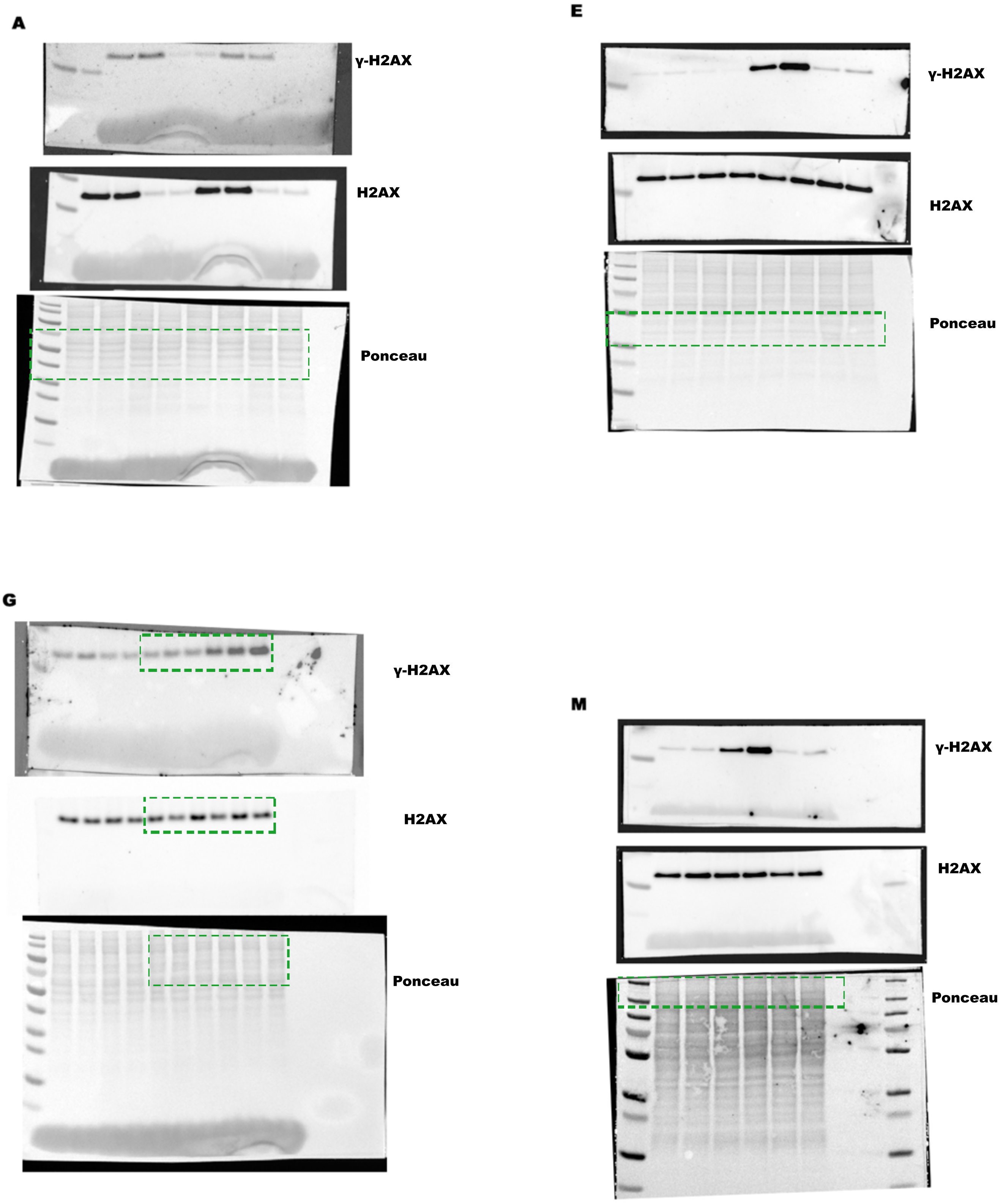
A
γ-H2AX
H2AX
Ponceau
E
γ-H2AX
H2AX
Ponceau
G
γ-H2AX
H2AX
Ponceau
M
γ-H2AX
H2AX
Ponceau

Figure 6

o

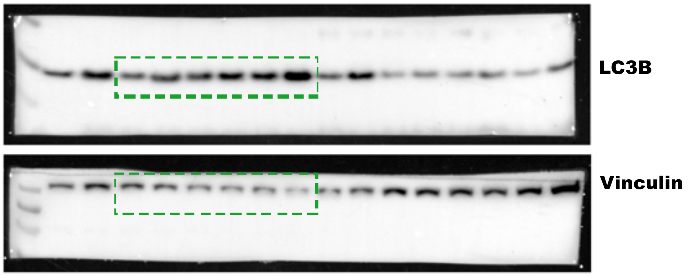

Figure 7

D

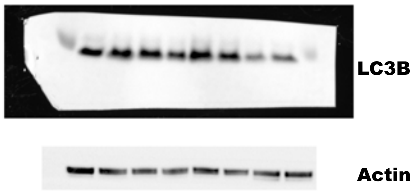

C

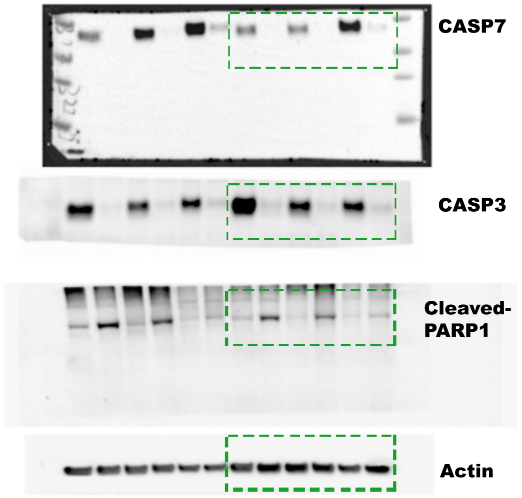

Supp_Figure 1

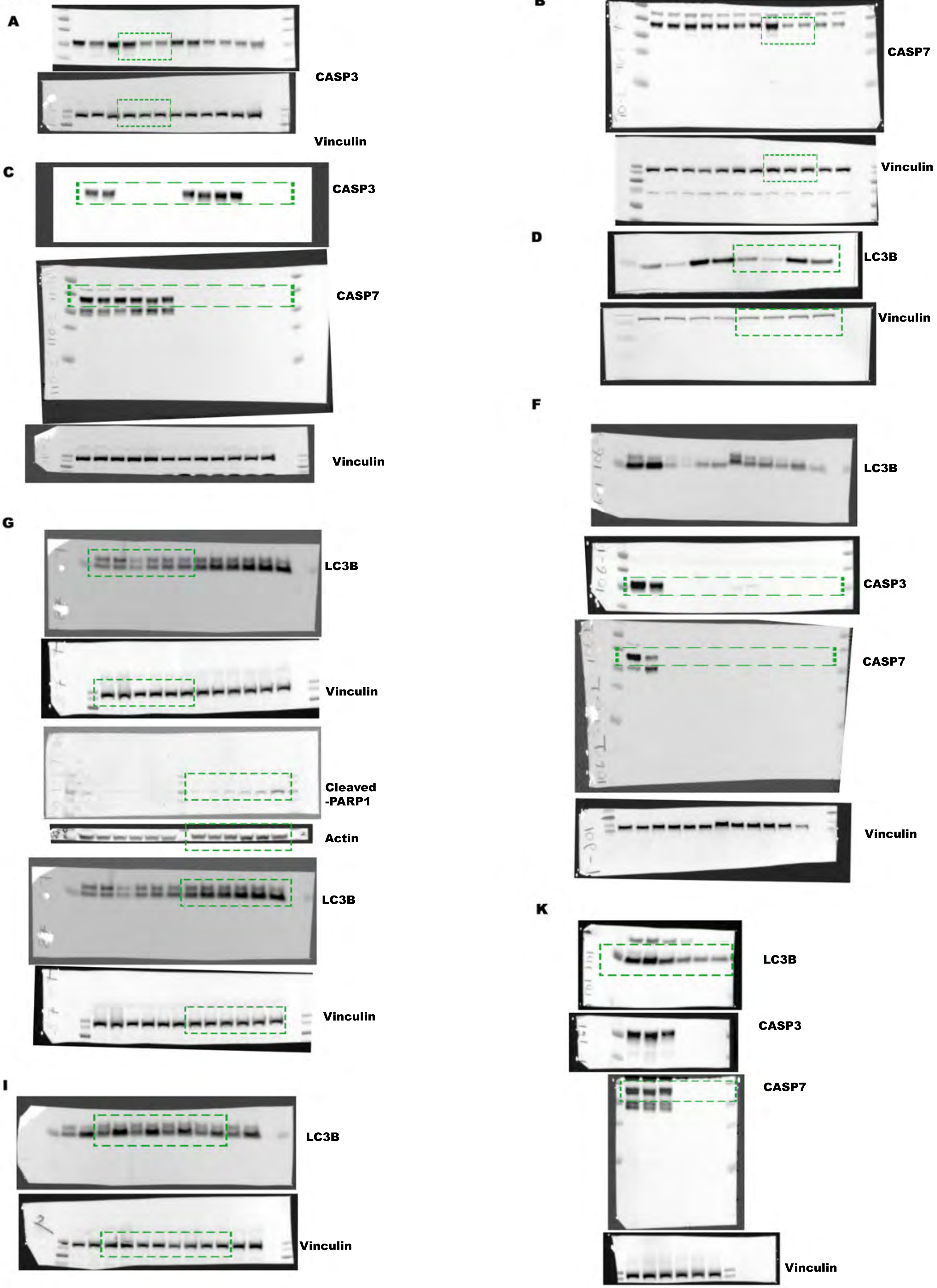

Supp_Figure 2

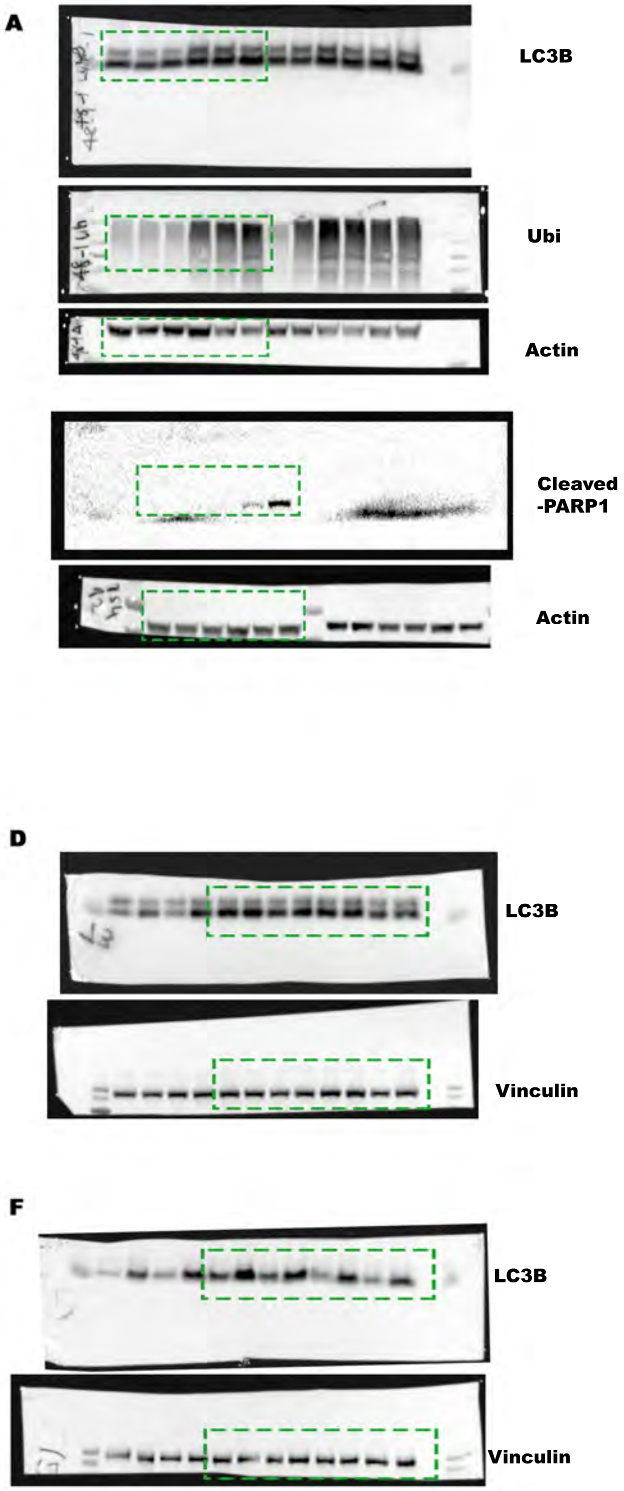

Supp_Figure 3

A

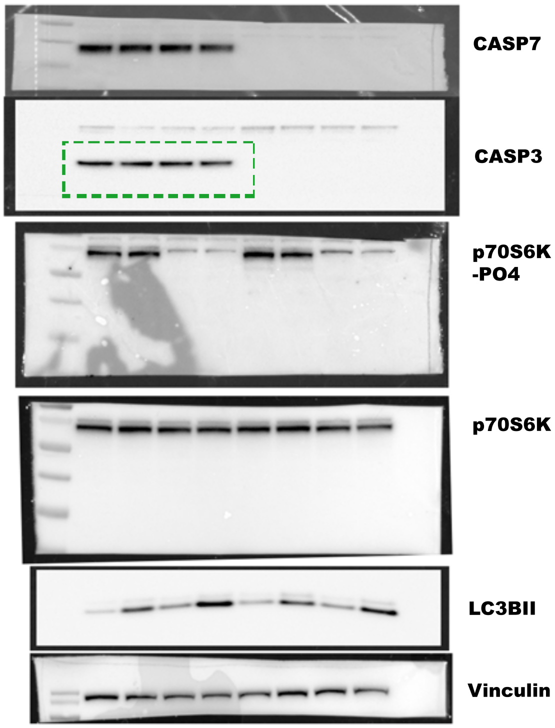

B

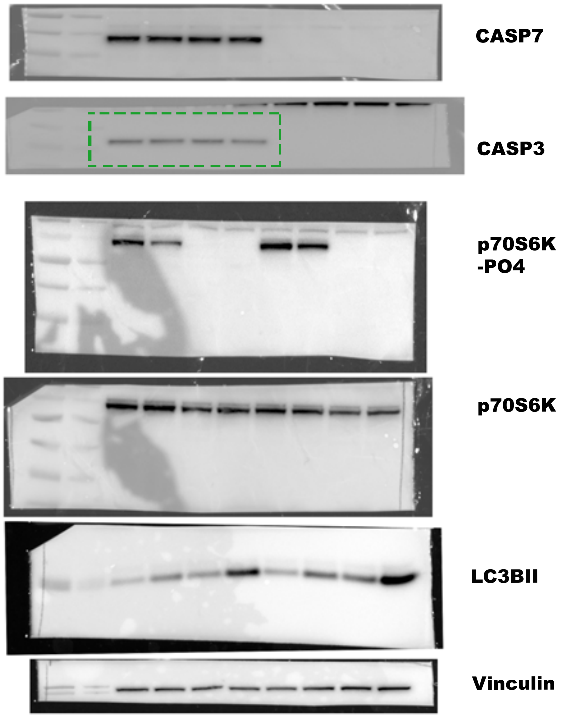

C

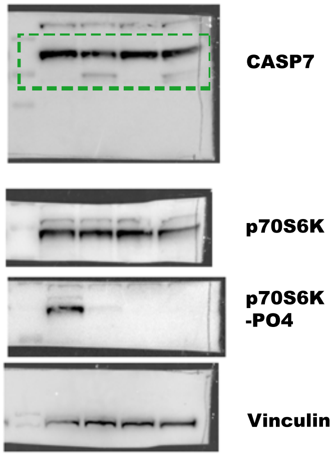

Supp_Figure 4

A

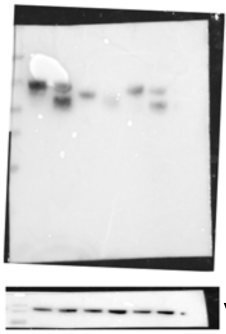

CASP7

Vinculin

B

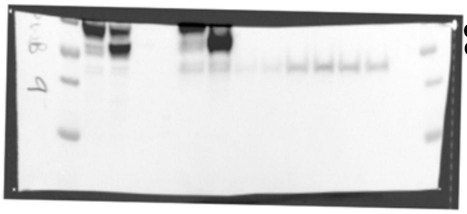

CASP7
CASP7-p29/30

D

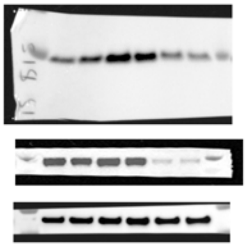

LC3B

calpain 2

Actin

Supp_Figure 5

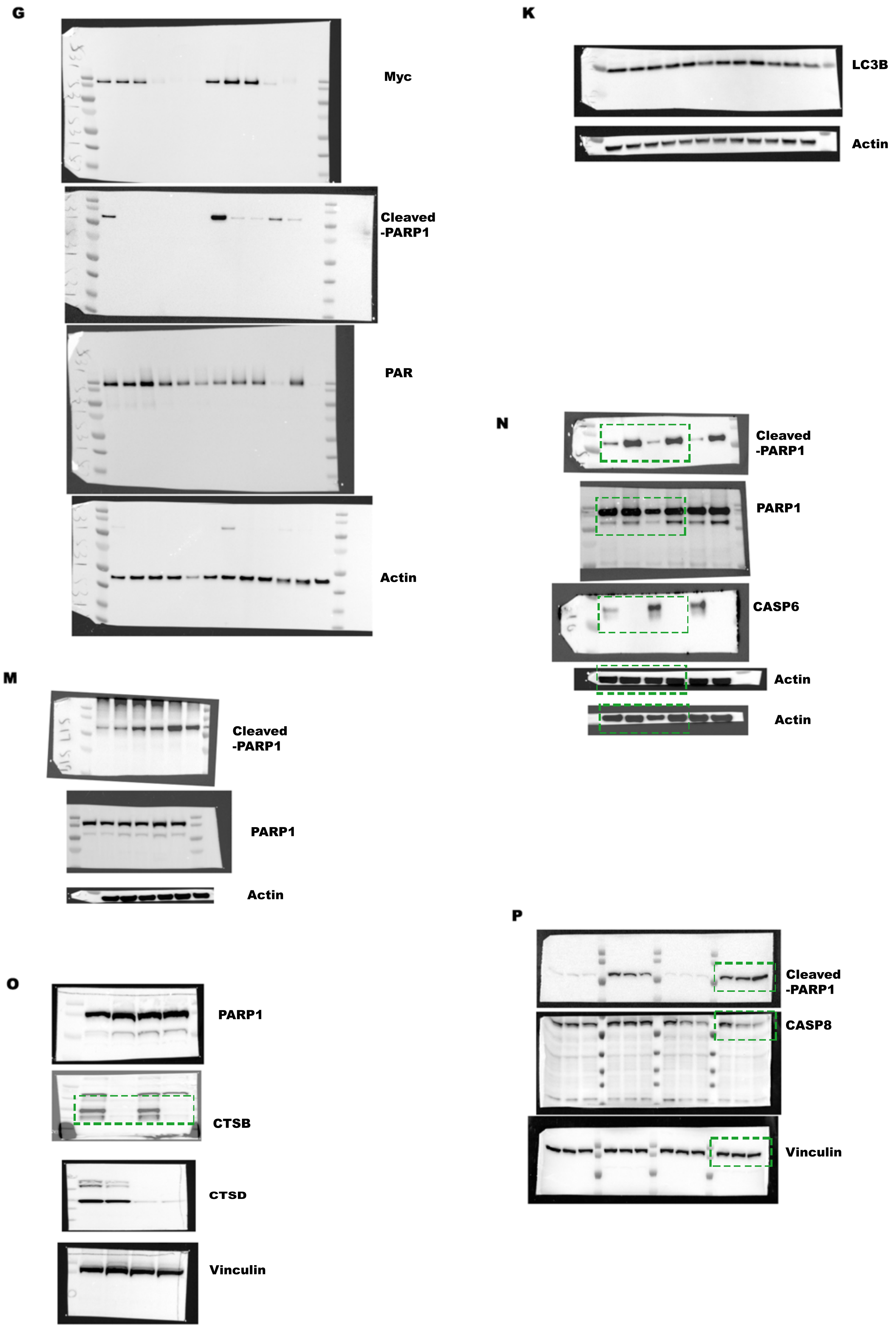

Supp_Figure 6

A

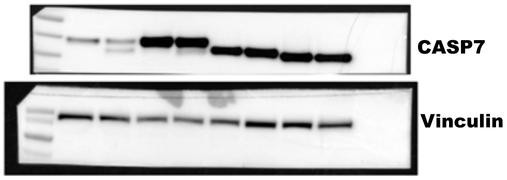

Supplement: S1 Raw Images — The green box indicates the area of the blot that was used for the figure when only a portion of the blot was used. Lanes have not been rearranged; the order of the lanes is same as the order shown in figures. (PDF) [file pbio.3003034.s008.pdf]
