# Supplementary material for: Caspase 3 and caspase 7 promote cytoprotective autophagy and the DNA damage response during non-lethal stress conditions in human breast cancer cells
Source: PLoS Biol. 2025 Feb 21;23(2):e3003034. doi: 10.1371/journal.pbio.3003034 (PMC11882052; doi:10.1371/journal.pbio.3003034)
Supplement: S1 Table — (DOCX) [file pbio.3003034.s011.docx]

| S1 Table: siRNAs used in knockdown experiments | |  |
| --- | --- | --- |
| siRNA | Source | Identifier |
| CASP3 siRNA 1 | Integrated DNA Technologies (IDT) | hs.Ri.CASP3.13.1-SEQ1 |
| CASP3 siRNA 2 | Integrated DNA Technologies (IDT) | hs.Ri.CASP3.13.1-SEQ2 |
| CASP7 siRNA 1 | Integrated DNA Technologies (IDT) | hs.Ri.CASP7.13.2-SEQ1 |
| CASP7 siRNA 2 | Integrated DNA Technologies (IDT) | hs.Ri.CASP7.13.2-SEQ2 |
| CASP2 siRNA 1 | Integrated DNA Technologies (IDT) | hs.Ri.CASP2.13.2 |
| CASP2 siRNA 2 | Integrated DNA Technologies (IDT) | hs.Ri.CASP2.13.9 |
| CASP8 siRNA 1 | Integrated DNA Technologies (IDT) | hs.Ri.CASP8.13.5 |
| CASP8 siRNA 2 | Integrated DNA Technologies (IDT) | hs.Ri.CASP2.13.8 |
| Cathepsin B siRNA | Integrated DNA Technologies (IDT) | hs.Ri.CTSB.13.1 |
| Cathepsin D siRNA | Integrated DNA Technologies (IDT) | hs.Ri.CTSB.13.2 |
| Scramble-siRNA  (control for CASP and Cathepsin siRNA experiments) | Integrated DNA Technologies (IDT) | DS NC1 |
| calpain1 siRNA | Santa-Cruz | sc-29885 |
| calpain2 siRNA | Santa-Cruz | sc-41459 |
| Scramble-siRNA  (control for calpain siRNA experiments) | Santa-Cruz | sc-37007 |
