# Supplementary material for: Caspase 3 and caspase 7 promote cytoprotective autophagy and the DNA damage response during non-lethal stress conditions in human breast cancer cells
Source: PLoS Biol. 2025 Feb 21;23(2):e3003034. doi: 10.1371/journal.pbio.3003034 (PMC11882052; doi:10.1371/journal.pbio.3003034)
Supplement: S2 Table — (DOCX) [file pbio.3003034.s012.docx]

| S2 Table: Forward and Reverse primer sequences used for qRT-PCR | |
| --- | --- |
| Primer Name | RT-qPCR Primers (Sequence 5' to 3') |
| ACTB-F | GCACAGAGCCTCGCCTT |
| ACTB-R | GTTGTCGACGACGAGCG |
| ATG5-F | TGATCCTGAAGATGGGGAAA |
| ATG5-R | TCCGGGTAGCTCAGATGTTC |
| ATG7-F | CGGGGGCAAGAAATAATG |
| ATG7-R | CCCAACATCCAAGGCACTAC |
| ATG12-F | TTGTGGCCTCAGAACAGTTG |
| ATG12-R | CCATCACTGCCAAAACACTC |
| LC3B-F | GAACGATACAAGGGTGAGAAGC |
| LC3B-R | AGAAGGCCTGATTAGCATTGAG |
| ULK1-F | CGTTGCAGTACTCCATAACCAG |
| ULK1-R | GGGGAAGGAAATCAAAATCC |
