# Supplementary material for: Caspase 3 and caspase 7 promote cytoprotective autophagy and the DNA damage response during non-lethal stress conditions in human breast cancer cells
Source: PLoS Biol. 2025 Feb 21;23(2):e3003034. doi: 10.1371/journal.pbio.3003034 (PMC11882052; doi:10.1371/journal.pbio.3003034)
Supplement: S3 Table — (DOCX) [file pbio.3003034.s013.docx]

| S3 Table: KEY RESOURCES TABLE |  |  |
| --- | --- | --- |
| REAGENT OR RESOURCE | SOURCE | IDENTIFIER |
| Antibodies and concentrations used |  |  |
| Mouse anti-β actin (1:500) | Abcam | 3700 |
| Rabbit anti-Bcl2 | Cell Signalling | 4223 |
| Rabbit anti-calpain1 (Mu) (1:1000) | Invitrogen | MA3-940 |
| Rabbit anti-calpain2 (1:1000) | Cell Signaling | 2539 |
| Rabbit anti-CASP2 | Proteintech | 10436-1-AP |
| Mouse anti-CASP8 | Proteintech | 66093-1-Ig |
| Rabbit anti-CASP3 (1:1000) | Cell Signaling | 9662 |
| Rabbit anti-CASP7 (1:1000) | Cell Signaling | 9492 |
| Mouse anti-CASP7 used in IP | LSBio | LS-C179785 |
| Rabbit anti-cleaved CASP3 (1:1000) (at ASp175) | Cell Signaling | 9661 |
| Rabbit anti-Cathepsin B | ProteinTech | 12216-1-AP |
| Rabbit anti-Cathepsin D | Cell Signalling | 2284 |
| Rabbit anti-FLAG (1:1000) | Sigma, | F7425 |
| Rabbit anti-H2AX (1:1000) | Abcam | 11175 |
| Rabbit anti-γH2AX (1:1000) | Abcam | 81299 |
| Rabbit anti-LC3B (1:1000) | Abcam | 48394 |
| Mouse anti-Myc-tag | Cell Signalling | 2276 |
| Rabbit anti-PAR | Cell Signaling | 83732 |
| Rabbit anti-PARP (1:1000) | Cell Signaling | 9542 |
| Rabbit anti-cleave-PARP (1:1000) (at Asp214) | Cell Signaling | 9541 |
| Rabbit anti-p70S6K | Cell Signaling | 2708 |
| Rabbit anti phosopho-p70S6K (Thr 389) | Cell Signaling | 9205 |
| Mouse anti-Ubi (1:1000) | Millipore | 04-263 |
| Rabbit anti-vinculin (1:1000) | Abcam | 129002 |
| Mouse IgG–HRP anti | Cell Signaling | 7076 |
| Mouse-IgG (used in IP) | Santa Cruze | Sc2025 |
| Rabbit IgG–HRP anti | Cell Signaling | 7074 |
| Rabbit Alexa Flour 568 | Invitrogen | A11011 |
| Media, Pharmacological Drugs, Chemicals, Peptides, and Recombinant Proteins |  |  |
| Agarose | Invitrogen | 16500-100 |
| Bafilomycin A 1 (BafA1) | Sigma | B1793-10UG |
| BCA Protein Assay Kit | Invitrogen | 23227 |
| Blocking buffer | LI-COR | 927-60003 |
| Bolt LDS sample buffer 4X | Invitrogen | B0007 |
| Bortezomib | Apexbio | A2614 |
| Complete mini protease inhibitor cocktail | Roche | 4693124001 |
| Crystal violet | Sigma | C6158 |
| DALRed (D677-10) | Dojindo | NC1879567 |
| DAPGreen (D676-10) | Dojindo | NC18879566 |
| DAPI | Thermo Fisher Scientific | D3571 |
| DDT | Sigma | 43816-10ML |
| Dulbecco’s Modified Eagle Medium (DMEM) | Gibco | 11995-065 |
| DMSO (Dimethylsulfoxide) | Fisher BioReagents | BP231-100 |
| Dnase 1(Amplification Grade) | Invitrogen | 18068-015 |
| Dynabeads Protein G | Thermo Fisher Scientific | 10003D |
| Earle’s Balanced Salt Solution (EBSS) | Sigma | E3024 |
| ECL Prime Western Blotting Detection Reagent (Clarity Western ECL) | BioRad | 170-5061 |
| e-Myco PCR Mycoplasma Detection Kit | Boca Scientific Inc | 25,235 |
| Ethanol | Commercial Alcohols | P016EAAN |
| F12 medium | Gibco | 11765-054 |
| Femto (SuperSignal West Femto Maximum Sensitivity Substrate | Thermo Fisher Scientific | 34095 |
| Fetal Bovine Serum (FBS) (Heat inactivated) | Invitrogen | 12,483,020 |
| Gels (4-12% Bolt Bis-Tris Plus) | Invitrogen | NW04125BOX |
| Gels (10% Bolt Bis-Tris Plus) | Invitrogen | NW00105BOX |
| GelRed | Biotium | 41003 |
| Geneticin (G418) | Invitrogen | 10131-035 |
| HEPES | Gibco | 15,630–080 |
| Hydrocortisone | Sigma | H4001 |
| Hygromycin | Invitrogen | 10687010 |
| Insulin | Gibco | 12585-014 |
| Lipofectamine 3000 | Invitrogen | L3000-008 |
| Lipofectamine RNAiMAX^TM^ | Invitrogen | 13778075 |
| MEM non-essential amino acids (MEM NEAA) | Gibco | 11140-050 |
| Methanol | Thermo Fisher Scientific | BPA4124 |
| MG132 | Apexbio | A2585 |
| Nupage LDS Buffer 4X | Invitrogen | NP0007 |
| NuPAGE™ MES SDS Running Buffer | Invitrogen | NP0002 |
| Nupage Transfer Buffer | Invitrogen | NP0006 |
| Olaparib | Selleckchem | S1060 |
| One-Step Plus SYBR Green reagent kit | Applied Biosystems | 4385617 |
| Opti-MEM | Gibco | 31985070 |
| PBS (1X) | Gibco | 10010-023 |
| Phage Ruler - preastain protein ladder | Thermo Fisher Scientific | 8159600147 |
| PhosSTOP | Roche | 4906845001 |
| PMSF (phenylmethylsulfonyl fluoride) | Santa-Cruz | SC24948 |
| Protease inhibitor cocktail | Santa-Cruz | SC24948 |
| Puromycin | Sigma | P6920 |
| PVDF membranes | BioRad | 1620177 |
| Rapamycin | Invitrogen | PHZ1235 |
| RIPA lysis buffer | Santa-Cruz | SC24948 |
| Rneasy Plus Mini Kit | QIAGEN | 74104 |
| Scramble-siRNA (control siRNA for calpain siRNAs | Santa-Cruz | sc-271313 |
| Scramble-siRNA (NCI) (control siRNA for CASP siRNAs) | Integrated DNA Technologies (IDT) | 51-01-14-04 |
| Skim milk | Commercial suppliers | NA |
| Slowfade Gold Antifade Reagent with DAPI | Thermo Fisher Scientific | 36939 |
| Sodium orthovanadate | Santa-Cruz | SC24948 |
| Staurosporine | Sigma | S6942 |
| Triton-X-100 | Sigma | x-100 |
| Trypsin | Gibco | 25300-062 |
| Critical Commercial Assays |  |  |
| NE-PER^TM^ Nuclear Cytoplasmic Extraction Reagent kit | Thermo Fisher Scientific | 78833 |
| Pierce IP Lysis Buffer | Thermo Fisher Scientific | 87787 |
| Proteasome-Glo Cell-Based Reagents kit | Promega | G862112 |
| Quick change II XL site directed mutagenesis kit | Agilent | 200521 |
| Bacterial Strains |  |  |
| One Shot Stbl3 chemically competent E. coli cells | Thermo Fisher Scientific | C737303 |
| Plasmids and Constructs |  |  |
| N-FLAG-BCL2 | Addgene | 18003 |
| CASP7 constructs (CASP7-WT) | Dr. Salvesen (Denault and Salvesen, 2003) | NA |
| CASP7 constructs (CASP7-NC) | Dr. Salvesen (Denault and Salvesen, 2003) | NA |
| CASP7 constructs (CASP7-deltaPro) | Dr. Salvesen (Denault and Salvesen, 2003) | NA |
| CASP7 constructs (CASP7-C186A) | Dr. Salvesen (Denault and Salvesen, 2003) | NA |
| CASP7 constructs (CASP7-CCM) | Made in this study | NA |
| CASP7 constructs (CASP7-p30) | Made in this study | NA |
| CASP7 constructs (CASP7-p29) | Made in this study | NA |
| CASP3 constructs CASP3-WT (clone #) | Sino Biological | HG10050-CH |
| N-Myc-PARP1-WT | SinoBiological | HG11040-NM |
| N-Myc-PARP1-DEVA | Made in this study | NA |
| N-Myc-PARP1-AEVA | Made in this study | NA |
| N-Myc-PARP1-CI (H862A, Y896A, and E988A) | Made in this study | NA |
| pcDNA3 plasmids | Invitrogen | V1020-20 |
| PX458 | Addgene | plasmid # 48138 |
| PX459 | Addgene | plasmid # 62988 |
| Cell lines , Tissues and PDX |  |  |
| JIMT1 | German Collection of Microorganisms and Cell Culture | ACC 589 |
| MDA-MB-231 | (American Type Culture Collection, ATCC) | CRM-HTB-26 |
| SKBR3 | (American Type Culture Collection, ATCC) | HTB-30 |
| SUM149-PT (BRCA1 mutant) | BioIVT | HUMANSUM-0003004 |
| SKBR3 and MDA-MB-231 cells stably transfected with CASP3 and/or 7 constructs | Made in this study | NA |
| Murine tissue | Dr. Nancy dos Santos | NA |
| PDX | Dr. Aparicio | NA |
| CRISPR gRNA Sequences |  |  |
| CASP3 gRNA1 AATGGACTCTGGAATATCCC TGG | NA | NA |
| CASP3 gRNA2 ATGTCGATGCAGCAAACCTC AGG | NA | NA |
| CASP7 gRNA ATGGCATCCAGGCCGACTCGGGG | NA | NA |
| Software |  |  |
| Adobe Photoshop CC | Adobe Systems | NA |
| CometScore 2.0 | (Robison et al., 2005) | NA |
| CRISPOR.org | Concordet and Haeussler, 2018) | NA |
| Deepcalpain Algorithm | (Liu et al., 2019) | NA |
| GraphPad Prism version 7.0 | www.graphpad. com | NA |
| GRETTA (v0.99.2) | (Takemon and Marra, 2022) | NA |
| ImageJ64 | https://imagej.nih.gov/ij/ | NA |
| Image Lab software (5.1) | Bio-Red | NA |
| R statistical software (v4.2.2) | (R Core Team 2020) | NA |
| Zen 2.5 | Zeiss | NA |
| BioRender | https://www.biorender.com/ | N/A |
