# Supplementary material for: Caspase 3 and caspase 7 promote cytoprotective autophagy and the DNA damage response during non-lethal stress conditions in human breast cancer cells
Source: PLoS Biol. 2025 Feb 21;23(2):e3003034. doi: 10.1371/journal.pbio.3003034 (PMC11882052; doi:10.1371/journal.pbio.3003034)
Supplement: Attachment — Submitted filename: Samarasekera_et_al_Response_to_Reviews_Nov19_2024_auresp_3.pdf [file pbio.3003034.s017.pdf]

Response to Reviews for PBIOLOGY-D-24-00592R1

We thank the Editor and the reviewers for taking the time to review our manuscript and provide insightful and constructive comments. We have carefully considered all of the comments, and our point-by-point response is provided below.

Please note that new text and revised text are indicated in red font in the "marked up" version of the manuscript. We modified the manuscript title (from "Caspase 3 and caspase 7 promote adaptation to non-lethal stress through Parp1" to "Caspase 3 and caspase 7 promote adaptation to non-lethal stress and their dual loss phenocopies Parp1 inhibition") to better reflect the findings presented in the manuscript.

Editor:

"As you will see below, the reviewers are generally positive and think that your study is interesting and well done. However, the reviewers raise overlapping concerns about the over-interpretation of some of the results and the level of mechanistic insight into how caspase-3/7 prevents PARP cleavage."

We have corrected the instances of over-interpretation of results in the revised manuscript. Details are provided in the response to Reviewers below.

We have added new data that provides mechanistic insights regarding the relationship between caspase-3/7 and PARP cleavage. Interestingly, our data indicate that caspase-2, at least in part, is responsible for cleaving PARP 1 in the absence of CASP3 and CASP7 (details in R#1, comment 4).

"Both Reviewers #1 and #3 suggest an experiment using a PARP cleavage-resistant mutant to see if it can rescue the caspase3/7 knockout phenotype".

We created PARP cleavage-resistant mutants (DEVD site) and evaluated these alongside PARP wild-type, PARP-catalytically inactive mutant and vector-only controls. The PARP DEVD variants were resistant to the excess cleavage typically observed in the CASP3+7 DKO background and showed a partial rescue of some, but not all, of the phenotypes (details in R#1, comment 6).

"In addition, Reviewer #2 raises concerns that it is not clear how Caspase-3/7 are being activated in the model and notes that it is important to investigate the potential role of the MOMP pathway".

We investigated the role of the MOMP pathway and have now demonstrated that MOMP is not involved in non-canonical activation of CASP7 in the non-lethal stress conditions examined (details in R#2 comment 1).

Reviewer #1:

Gayathri Samarasekera and colleagues have submitted a manuscript titled "Caspase 3 and caspase 7 promote adaptation to non-lethal stress through PARP1" for review. The authors propose that unconventional and non-apoptotic functions of Caspase-3 and -7 are essential for stress adaptation, mediated by Parp1 modulation affecting autophagy and the DNA damage response. While some sections present convincing data, such as alternative cleavage of Casp-3 and -7 under non-lethal stress, and dependency on calpains for this cleavage, other results are occasionally overinterpreted and confusing. The rationale for experimental design and result descriptions is unclear in several sections, and it is uncertain whether the effects described are specific to tumour cells or have broader biological significance. Additional experimental work and clearer organisation and description of the text are needed prior publication. Specific points for the authors to address are outlined below.

We expect that the cytoprotective effects described are not specific to tumor cells but have wide biological relevance since we detected CASP7-p29/30 fragments in multiple tissue samples obtained from healthy mice. Further, our previous study which demonstrated caspase dependency in cytoprotective autophagy was conducted in normal tissues of a Drosophila model. These points were clarified on p. 12 and 27.

1. The authors should consider demonstrating the autophagy effects 24 hours post-starvation in caspase-deprived cells without Bafilomycin treatment, even if the resolution of the experiment is less clear.

We evaluated autophagy effects at 24 hours post-starvation in CASP3+7 double knockout (DKO) cells with and without BafA1 treatment. In the absence of BafA1, there was a significant reduction of LC3B-II levels in starvation conditions. This reduction could be a reflection of either reduced autophagy or enhanced autophagy (ie. increased turn-over of LC3B-II). To distinguish these possibilities, we evaluated autophagy flux, ie. LC3B-II levels in the presence of BafA1. Consistent with the effects observed after 8 hours starvation, we found that there was no increase in autophagic flux following 24 hours starvation. New figure panels S1D and S1E were added to Figure S1 and the relevant text was added to pg. 8. The methods section was updated on pg. 29.

2. The conclusions drawn from Figure 1I-J are problematic because the number of Dalgreen dots per cell, used as a proxy for autophagy, is not normalized for cell size. Given the significantly reduced size of double Casp-KO cells, it is unclear how the authors conclude that there is less autophagy in absolute terms.

To address this concern, we have now added phase contrast images of parental and caspase DKO cells and quantified cell size. As demonstrated in new Figures S1M and S1N, there is no significant difference in cell size between the two cell lines. Thus, the reduction in number of autolysosomes in CASP3+7 DKO cells was not due to a difference in cell size. This important point was emphasized in the text on pg. 9 and the methods section was updated on pg. 38-39.

3. Regarding Figure 4C, the rationale for creating Casp7 calpain-resistant mutants using multiple amino acid substitutions instead of a single S>A conversion is unclear. This approach could unpredictably alter the general folding of the caspase template. Additionally, why the generation of the -29/30 fragments is not fully abolished in these mutants (as shown in the Western blot in Figure 4C) should be addressed.

Calpains are known to recognise the three dimensional structure rather than the sequence of the target site of the substrate protein (Tompa et al., 2004; Liu et al., 2019). It was shown previously that a single amino acid mutation of a target sequence does not prevent the recognition by calpains (Gafni et al., 2004; Gafni et al., 2009). Based on this prior work, we sequentially altered amino acid residues at the predicted calpain cleavage sites on CASP7 until a significant reduction (though not complete as noted by the reviewer) in the CASP7 non-canonical cleavage was obtained. We have updated the methods section and included the relevant references to provide the rationale for our approach (on pg. 35).

4. *Starting from Figure 5, the text becomes unclear. The authors suggest that the autophagy effects are somehow related to Parp1. How Parp1 is cleaved in the absence of Casp-3 and -7 needs to be elucidated.*

Based on our findings of the altered Parp1 exosite in CASP7 fragments, and previous reports linking Parp1 activity to autophagy regulation (Lee et al., 2016; Wang et al., 2018; Rodríguez-Vargas et al., 2019), we proposed that the autophagy effects in the non-lethal context examined here may also be related to Parp1. We clarified this rationale on p. 15-16. To address the question of Parp1 cleavage in the absence of CASP3+7, we first investigated the effector caspase CASP6 as well as several proteases (calpain1, calpain2, cathepsin B/D) that were shown previously to be capable of PARP proteolysis (Bhaskara et al., 2009;Chaitanya et al., 2010). We found that the siRNA-mediated knockdown of these proteases did not reduce Parp1 cleavage, but instead enhanced Parp1 cleavage, in the absence of CASP3 and 7.

We next investigated caspase 2 and caspase 8, both shown to localize to the nucleus in some conditions (Benchoua et al., 2002; Baliga et al., 2003). We discovered that knockdown of CASP2, but not CASP8, reduced the PARP1 cleavage in CASP3+7 DKO cells. Given that we used a siRNA approach to reduce protease activity, we acknowledge that we cannot definitively rule out other proteases, but our results point to a role for CASP2. In line with this finding, we also detected a significant increase in CASP2 levels in CASP3+7 DKO cells relative to parental cells under control (scramble-siRNA) conditions.

We also evaluated PARylation and autophagy effects following CASP2 knockdown in the CASP3+7 DKO cells. Despite the reduction in Parp1 cleavage, we did not detect recovery of PARylation or autophagic flux in CASP3+7 DKO cells though future studies using alternate approaches to enhance KD or KO of CASP2 are warranted (see also response to comments 5 and 6 below).
New figure panels 5J-O and S5M-P were added with related text on p. 16-17 and updated methods on p. 32.

5. The authors mention that excess Parp1 cleavage compromises Parp-1 activity. Is a catalytically dead version of Parp1 sufficient to prevent the autophagy effects observed in Casp-3-7 KO cells?

PARylation is a PARP catalytic activity-dependent process (Spiegel et al., 2018), and PARP and PARylation were demonstrated to play roles in the induction of autophagy (Rodríguez-Vargas et al., 2012; Lee et al., 2016). Thus, we predict that a catalytically dead version of PARP would result in a reduction in both PARylation and autophagy induction. In response to the reviewer's question, we made catalytically inactive PARP constructs (PARP-CI) by sequentially mutating three amino acid residues (H862, Y896, and E988) in the catalytic (CAT) domain of PARP (Spiegel et al., 2018). Previous studies showed that these alterations result in dominant negative activity (Zhengping et al., 2023). However, consistent with reported toxicity and embryonic lethality (Zhengping et al., 2023), the ectopic expression of PARP-CI in our cell lines resulted in cell death. In the surviving cells, there was detectable expression of PARP-CI but at a relatively low level compared to the PARP-WT construct expression (New figure panels S5G-L).

As expected, we observed significantly reduced PARylation in the PARP-CI background (Figure S5G and S5I). However, this observation may simply be a result of differences in PARP expression levels. We did not observe reduced autophagic flux (LC3BII levels) in the PARP-CI background as originally expected but note that an increase in autophagic flux is likely to occur as a stress response to cell death (Ikeda et al., 2022' Nah et al., 2022; Liu et al., 2023).

To address these issues, we attempted to create cell lines stably expressing PARP-CI, PARP-WT and PARP-DEVD site mutants (comment 6 below) in both SKBR3 and MDA-MB-231, but the cells either died or did not stably retain the expression constructs (see also comment 6).
New data are in Figures S5G-L, new text on pg. 15-16 and updated methods on pg. 34 and 36.

Overall, the results regarding the relationship of PARylation and autophagy effects to PARP-cleavage are inconclusive. Since we cannot conclude that these effects are occurring "through" PARP1, we have revised our interpretations (pg. 16, 17 and 25) and the manuscript title accordingly.

6. According to the authors' results, a non-cleavable caspase version of Parp1 would be expected to sustain Poly(ADP-ribosyl)ation and autophagy gene transcription. Is this prediction accurate?

To investigate this question, we made non-cleavable PARP constructs mutating the DEVD consensus sequence on PARP1 (PARP1-DEVA and PARP1-AEVA) and compared them with the PARP1-WT overexpression, PARP1-CI and vector only control in parental and in DKO cells.
Relative to the PARP1-WT control, both PARP1-DEVA and PARP1-AEVA demonstrated blocked cleavage in CASP3+7 DKO cells, confirming that the protease(s) capable of cleaving PARP1 in the absence of CASP3+7 is a protease that targets the DEVD site (Figures S5G and S5H). This result is in alignment with identification of CASP2 (comment 4).

PARP1-WT overexpression in CASP3+7 DKO cells resulted in a significant accumulation of cleaved-PARP1 fragments (89KD) compared to the PARP1-WT overexpression in parental cells, consistent with the increase in endogenous PARP cleavage we observed in the absence of CASP3+7 (Figures 5A-5D).

Expression of any of the PARP1 constructs (WT, DEVA, AEVA) increased the PARylation in both parental and DKO cells compared to the catalytically inactive PARP1 construct (CI). However, we also detected increased PAR in the vector control which may be due to the stress of transient transfection. To address this, we attempted to make stably expressing cell lines, but again were unsuccessful.

Lastly, as suggested by the reviewer, we investigated the effects on autophagy gene transcription using QRT-PCR of LC3B (Figure S5J). We detected an increased trend of LC3B transcript levels in PARP1-DEVA and PARP1-AEVA relative to PARP-WT and vector control. This trend is consistent with our prediction, but the issues with reporter expression level differences and induction of cell death (comment 5) remain.

The association between PARP-cleavage and LC3B transcription remains consistent, but we cannot conclude that these effects are occurring only "through" PARP and have modified our statements accordingly (pg. 16, 17, 25, title).

7. Casp-3 and Casp-7 appear to act redundantly regarding Parp-1, but the specificity mechanisms for the effects on γ-H2AX are specific to Casp-7. The biological relevance of this phenotype and why only double deficiency in Casp3 and Cas-7 can mimic the effects of Parp1 deprivation should be discussed. Additionally, it is intriguing that, as depicted in the model Figure, both Caspase-3 and -7 are required for all the phenotypes, even though Parp-1 cleavage is mainly associated with Caspase-7. Is there an additive effect in the absence of Caspase-3 when eliminating Caspase-7 activity?

We agree that the effect of CASP3 and CASP7 on PARP-1 appears to be redundant. We observed this redundancy or partial redundancy not only with PARP1, but with γ-H2AX and autophagy as well. We included in the text (pg. 18 and 25) that the effect on γ-H2AX is not specific to CASP7 but appears to be more dependent on CASP7 relative to CASP3 (Figure 6E and 6F). The γ-H2AX level was reduced in CASP 3+7 DKO cells but higher in both CASP3 and CASP7 single KO cells, with CASP3 single KO having a higher level of γ-H2AX compared to CASP7 single KO. Hence, the effect on γ-H2AX is also redundant and this explains the requirement for double deficiency in CASP3+7 to mimic the effects of Parp1 inhibition. We clarified this point in the Results on pg. 18 and the Discussion on pg. 25.

Reviewer #2*:*

In this manuscript by Samarasekera et al, the authors present a non-apoptotic function for caspases-3 and -7 in promoting stress induced cryoprotective autophagy. Overall this is an interesting paper and the concept of non-canonical cleavage of caspase-7 inducing this in the

absence of cell death is compelling. The experiments are well designed and the data presented is convincing. I only have a couple of suggestions

1.It is not clear how caspase-3 and -7 are being activated in this context. Is this through the mitochondrial pathway? It has been shown that GAPDH can promote autophagy and survival in cells that have undergone mitochondrial outer membrane permeabilization (MOMP) in the absence of caspase activity. While I don't think this is the same pathway, it would be good to rule in or out any effect of mitochondrial permeabilization in this context. Overexpression of Bcl2 or BclxL would be sufficient to address this to see if it mimics the caspase-7/3 knockout.

Thank you for this interesting suggestion. We overexpressed Bcl2 in SKBR3 parental cells and evaluated whether it affected non-canonical CASP7 processing (CASP7-p29/p30 formation in non-lethal stress). Bcl2 overexpression is reported to block MOMP and subsequent CASP3 and 7 activation in apoptosis (Llambi et al., 2011; Saddam et al., 2024). Accordingly, Bcl2 overexpression inhibited canonical caspase processing in staurosporine (STS)-induced apoptotic stress (new Figure 3F). In contrast, we found that Bcl2 overexpression did not inhibit the formation of CASP7-p29/30 in the non-lethal stress condition (4 h starvation) (Figure 3F). These results serve to rule out the involvement of MOMP in CASP7 activation in this context of non-lethal stress.

We have added text on pg. 11, included the new data in Figure 3F and updated methods in pg. 34.

2.In the calpain knockdown experiments in Figure 4 the results (F-I) do not appear to be statistically significant. Do these cells still express caspase-3? If this was done in a caspase-3 knockout/knockdown background the differences on LC3BII levels might be more extensive (assuming that caspase-3 is processed by a different mechanism)

The decrease in LC3B-II levels following calpain 2-siRNA treatment was statistically significant (relative to the scramble-siRNA control) in the MG132 non-lethal stress conditions only. In starvation conditions, LC3B-II levels were also decreased (but not statistically significant) in calpain 2-siRNA treated cells. In both conditions, the ratio of p29/30 to full length CASP7 was decreased (but not statistically significant) in calpain2-siRNA treated cells, but higher in calpain1-siRNA treated cells. These previous experiments were conducted in parental SKBR3 cells which contain CASP3, so we agree that it is a great idea to test in CASP3 KO/KD conditions.

We repeated the experiments using CASP3 KO cells but found that the effects on LC3B-II were not enhanced relative to the parental cells (new Figure S4D and S4E). This result is consistent with the possibility that both CASP3 and CASP7 might be affected in our calpain knockdown experiments. In fact, CASP3 was also reported to be processed by calpains (Blomgren et al., 2001;Nelson et al., 2012). Interestingly, we did observe a potential non-canonical processing of CASP3 (Figure 3). As mentioned in the manuscript, we did not follow CASP3 processing in this study as the data was not consistent across experiments, but this remains an interesting avenue for future investigation.

We have added text on pg. 14 in the revised manuscript and included the new data in Figure S4 (Figure S4D and S4E).

Reviewer #3: *Caspase 3 and caspase 7 promote adaptation to non-lethal stress through PARP1 Samarasekera et al.*

General comments

This is an interesting series of experiments that are well presented and clearly explained.

The authors make four main claims in the manuscript: (1) CASP3 and CASP7 promote cytoprotective autophagy induction in response to nonlethal stress; (2) CASP7 processing upon non-lethal stress is distinct from its processing in apoptosis; (3) PARP cleavage is increased and PARP1 activity and DNA Damage Response (DDR) signaling are reduced following CASP3 and CASP7 inhibition; and, (4) loss of CASP3 and CASP7 is synthetic lethal with loss of BRCA1.

Claim 1 is in two parts: cytoprotection and autophagy. Autophagy is studied with a range of assays for different endpoints and is convincing. The claim for cytoprotection relies on Figure 2J and 2K and a supplementary figure which show the effect of MG132 and Bortezomib in other cell types which show decreased viability in Casp7/7 DKO cells compared to wild-type cells. The data presented are clear, but there are no data showing cytoprotection for starvation. Are Casp3/7 DKO cells more vulnerable than wild type cells to starvation?

We quantified viability in post starvation stress in parental and in CASP3+7 DKD SKBR3 cells and observed a statistically significant reduction in cell viability in CASP 3+7 DKD cells at 72hrs following starvation. This data supports a role for CASP3+7 in cytoprotection in response to starvation stress. New figure panels S2N and S2O were added to Figure S2 and the relevant text was incorporated on pg. 10. The methods section was updated accordingly on pg. 37.

Claim 2 is supported by the data presented using a combination of approaches including rescue experiments in DKO cells. The authors link the non-cannonical cleavage to a previous report that calpain can cleave and activate caspase-7.

Claim 3 is supported by assays for PARP cleavage, PARylation and Histone 2X phosphorylation. The data are clear.

Claim 4 is supported by data comparing human tumor cell lines that have or lack BRAC1.

Overall the experiments are clearly described, the data are well presented and the authors' conclusions are clear. However, although the Casp3/7 DKO affects PARP, and inhibiting PARP or knocking out Casp3/7 sensitizes cells, how caspase-3/7 prevents PARP cleavage is unaddressed.

As detailed in R#1, comment 4, we investigated a number of possible candidate proteases and our data indicate that CASP2 plays a role in cleaving PARP1 in the absence of CASP3 and CASP7

(new Figures S5M-P and 5J-5O). Interestingly, CASP3 (Harvey et al., 1996) and CASP7 (Paroni et al., 2001) were shown to be capable of CASP2 cleavage, and we detected a significant increase in levels of CASP2 in the CASP3+7 DKO background relative to the parental cells (Figure 5L). Future studies are required to more fully elucidate the nature of CASP2 regulation by CASP3 and 7 in the context of non-lethal stress.

Comments on the Introduction

The authors summarize the evidence that caspases interact with components of autophagy in both mammalian and fly models, which includes both initiator and effector caspases. In the light of this summary, the meaning of the final sentence of the paragraph: "whether effector caspases have an evolutionarily conserved function is unknown" is not clear. I suggest emphasizing that mammalian initiators were known to interact with autophagy proteins, and that the current study investigated effectors.

We clarified this sentence as suggested (on pg. 5) and noted that initiator caspases have also not been systematically investigated in this context.

Comments on the experiments and the results section

The authors show evidence that calpain cleavage of caspase-7 and that the calpain cleavage is near PARP binding exosite in caspase-7. However the p30 form (with exosite) and p29 (lacking exosite) have similar functions and the exosite may therefore not be relevant to the non-apoptotic effects of caspase-7. The authors also show that loss of caspase-7 increases PARP cleavage and decreases PARP activity. In addition, they link PARP activity to the expression of a couple of autophagy genes. The authors link these observations to cancer by showing synthetic lethality between BRCA1 and the loss of caspase.

There is no further explanation of the mechanism of (1) the PARP cleavage seen in the DKO cells or (2) how caspase-7 suppresses PARP cleavage. The authors show that loss of caspase affects PARP and loss of caspases mimics PARP inhibition and conclude that caspases "promote adaptation through PARP" (my underlining). The authors' claim is very strong and I agree that it seems likely that caspase act through PARP, but the authors didn't test this idea. For example, does overexpression of a PARP mutant that cannot be cleaved rescue the caspase DKO phenotype?

Please see response to R#1, comment 6.

Comments on the Discussion

I think the authors could be clearer about their position, re-writing some sections or by providing experimental evidence if they have it.

1. Use of "differentially" is unnecessary in "CASP7 is differentially cleaved, resulting in stable fragments (CASP7-p29/30) that are distinct from canonical cleaved-CASP7 fragments (CASP7-p20/p12) reported in apoptosis."

The word "differentially" was removed from the sentence (pg. 22).

2. The statement: "Overall, this study shifts the current paradigm of apoptotic caspase-PARP1 interactions to one that involves non-canonical caspase cleavage and the promotion of cell adaptation and survival pathways at the onset of cellular stress or in non-lethal cellular stress conditions" is a key claim of the authors. I take interactions to mean binding, but do the authors mean that PARP binds Caspase-7 activated by non-canonical cleavage? Later in the text the authors write:

"Since CASP7-p29 does not have an intact exosite, one would expect (my underlining) it to differently modulate PARP1 and downstream stress response pathways. Despite this key difference, expression of either exogenous p29 or p30 alone were able to enhance the autophagy response or the DNA damage response (γ-H2AX), revealing a potential functional similarity between these non-canonical CASP7 fragments." As one fragment that might bind PARP and one that cannot exert similar effects isn't this evidence that the effects of the non-canonical fragments are independent of PARP binding?

We agree with the Reviewer that the effects of the non-canonical fragments are likely independent of PARP binding. We also acknowledge that both fragments may be unable to bind PARP. We have clarified our position and revised the text on pg. 22-23.

"Since CASP7-p29 does not have an intact exosite, we initially expected it to differently modulate PARP1 and downstream stress response pathways. Despite this key difference, expression of either exogenous p29 or p30 alone was able to enhance the autophagy response or the DNA damage response (γ-H2AX), revealing a potential functional similarity, which is likely independent of PARP1 binding, between these non-canonical CASP7 fragments."

3. Other specific question to be more clearly addressed include:

Non-canonical cleavage of caspase-7 is reported to increase the activity of caspase-7, how does increased activity fit with a non-apoptotic outcome?

We have now addressed this question on pg. 23:
"However, the p29 fragment lacking the PARP1 binding exosite shows the significantly highest level of LC3B-II flux or γ-H2AX. This result is somewhat consistent with an in-vitro cell-free analysis that demonstrated higher enzymatic activity when CASP7 cleavage occurs at the second calpain cleavage site (equivalent to the p29 site), compared to the p30 or canonical cleavage sites (Gafni et al., 2009). In that study, however, the initial processing was followed by a second cleavage event to generate the enzymatically active tetrameric complex composed of

p17 and p12 fragments (Gafni et al., 2009b). In non-lethal contexts, we predict that the retention of CASP7-p30 and/or p29 forms serves to limit apoptotic activity."

Comments on the Figures and Figure legends

Typographical error Figure 6M. Two periods at the sentence's end.

The typographical error was corrected (pg. 68).

Summary

This is a very interesting manuscript, with well performed experiments that are clearly presented. It is very well suited to PLOS Biology. The major point the authors should address experimentally is whether an "uncleavable" PARP can rescue the DKO phenotype. This opinion is the basis of the decision "Major Revision". The other points can be addressed by revising the indicated areas of text.

Thank you; the text was revised in multiple sections, and please see R#1, comment 6 for a detailed response regarding the uncleavable PARP construct.

New References added:

Baliga, B. C., Colussi, P. A., Read, S. H., Dias, M. M., Jans, D. A. and Kumar, S. (2003). Role of prodomain in importin-mediated nuclear localization and activation of caspase-2. *J. Biol. Chem.* **278**, 4899–4905.

Benchoua, A., Couriaud, C., Guégan, C., Tartier, L., Couvert, P., Friocourt, G., Chelly, J., Murcia, J. M. De and Onténiente, B. (2002). Active caspase-8 translocates into the nucleus of apoptotic cells to inactivate poly(ADP-ribose) polymerase-2. *J. Biol. Chem.* **277**, 34217–34222.

Bhaskara, V., Challa, S., Panigrahi, M. and Babu, P. (2009). Differential PARP cleavage: An indication for existence of multiple forms of cell death in human gliomas. *Neurol. India* **57**, 264–268.

Blomgren, K., Zhu, C., Wang, X., Karlsson, J. O., Leverin, A. L., Bahr, B. A., Mallard, C. and Hagberg, H. (2001). Synergistic activation of caspase-3 by m-calpain after neonatal hypoxia-ischemia: A mechanism of "pathological apoptosis"? *J. Biol. Chem.* **276**, 10191–10198.

Chaitanya, G. V., Alexander, J. S. and Prakash Babu, P. (2010). *PARP-1 cleavage fragments: signatures of cell-death proteases in neurodegeneration*.

Gafni, J., Hermel, E., Young, J. E., Wellington, C. L., Hayden, M. R. and Ellerby, L. M. (2004). Inhibition of calpain cleavage of Huntingtin reduces toxicity: Accumulation of calpain/caspase fragments in the nucleus. *J. Biol. Chem.* **279**, 20211–20220.

Gafni, J., Cong, X., Chen, S. F., Gibson, B. W. and Ellerby, L. M. (2009a). Calpain-1 cleaves and activates caspase-7. *J. Biol. Chem.* **284**, 25441–25449.

Gafni, J., Cong, X., Chen, S. F., Gibson, B. W. and Ellerby, L. M. (2009b). Calpain-1 cleaves and

activates caspase-7. *J. Biol. Chem.* **284**, 25441–25449.
Ikeda, S., Zablocki, D. and Sadoshima, J. (2022). The role of autophagy in death of cardiomyocytes. *J. Mol. Cell. Cardiol.* **165**, 1–8.
Lee, H. R., Gupta, M. K., Kim, D. H., Hwang, J. H., Kwon, B. and Lee, H. T. (2016). Poly(ADP-ribosyl)ation is involved in pro-survival autophagy in porcine blastocysts. *Mol. Reprod. Dev.* **83**, 37–49.
Liu, Z. X., Yu, K., Dong, J., Zhao, L., Liu, Z., Zhang, Q., Li, S., Du, Y. and Cheng, H. (2019). Precise prediction of calpain cleavage sites and their aberrance caused by mutations in cancer. *Front. Genet.* **10**, 1–13.
Liu, S. Z., Yao, S. J., Yang, H., Liu, S. J. and Wang, Y. J. (2023). Autophagy: Regulator of cell death. *Cell Death Dis.* **14**,.
Llambi, F., Moldoveanu, T., Tait, S. W. G., Bouchier-Hayes, L., Temirov, J., McCormick, L. L., Dillon, C. P. and Green, D. R. (2011). A Unified Model of Mammalian BCL-2 Protein Family Interactions at the Mitochondria. *Mol. Cell* **44**, 517–531.
Nah, J., Zablocki, D. and Sadoshima, J. (2022). The role of autophagic cell death in cardiac disease. *J. Mol. Cell. Cardiol.* **173**, 16–24.
Nelson, W. B., J, S. A., Hudson, M. B., Talbert, E. E. and Powers, S. K. (2012). Cross-talk between the calpain and caspase-3 proteolytic systems in the diaphragm during prolonged mechanical ventilation. *Crit Care Med.* **40**, 1857–1863.
Paroni, G., Henderson, C., Schneider, C. and Brancolini, C. (2001). Caspase-2-induced Apoptosis is Dependent on Caspase-9, but Its Processing during UV- or Tumor Necrosis Factor-dependent Cell Death Requires Caspase-3. *J. Biol. Chem.* **276**, 21907–21915.
Rodríguez-Vargas, J. M., Ruiz-Magãa, M. J., Ruiz-Ruiz, C., Majuelos-Melguizo, J., Peralta-Leal, A., Rodríguez, M. I., Muñoz-Gámez, J. A., De Almodóvar, M. R., Siles, E., Rivas, A. L., et al. (2012). ROS-induced DNA damage and PARP-1 are required for optimal induction of starvation-induced autophagy. *Cell Res.* **22**, 1181–1198.
Rodríguez-Vargas, J. M., Oliver-Pozo, F. J. and Dantzer, F. (2019). PARP1 and poly(ADP-ribosyl)ation signaling during autophagy in response to nutrient deprivation. *Oxid. Med. Cell. Longev.* **2019**,.
Saddam, M., Paul, S. K., Habib, M. A., Fahim, M. A., Mimi, A., Islam, S., Paul, B. and Helal, M. M. U. (2024). Emerging biomarkers and potential therapeutics of the BCL-2 protein family: the apoptotic and anti-apoptotic context. *Egypt. J. Med. Hum. Genet.* **25**,.
Spiegel, J. O., Houten, B. Van and Durrant, J. D. (2018). PARP1: Structural Insights and Pharmacological Targets for Inhibition. *Physiol. Behav.* **176**, 139–148.
Tompa, P., Buzder-Lantos, P., Tantos, A., Farkas, A., Szilágyi, A., Bánóczi, Z., Hudecz, F. and Friedrich, P. (2004). On the sequential determinants of calpain cleavage. *J. Biol. Chem.* **279**, 20775–20785.
Wang, C., Xu, W., Zhang, Y., Zhang, F. and Huang, K. (2018). PARP1 promote autophagy in cardiomyocytes via modulating FoxO3a transcription. *Cell Death Dis.* **9**, 1047.
Zhengping, S., Lee, B. J., Zhanga, H., Lina, X., Chen, L., Jianga, W., Napon, C., Steven, G., Shenb, M. M., Baera, R., et al. (2023). RESEARCH ARTICLE | GENETICS Inactive PARP1 causes embryonic lethality and genome instability. *Proc. Natl. Acad. Sci.* **120**, e2301972120.
